# The effects of plyometric training on adolescent sports performance: a systematic review and meta-analysis

**DOI:** 10.7717/peerj.21585

**Published:** 2026-07-23

**Authors:** Song Yuan, Yunmei Chai, Zhijun Tan, Zhibo Cui, Quanhong Lu, Jingmiao Wang

**Affiliations:** 1 Football Academy, Chengdu Sport University, Chengdu, China; 2 School of Physical Education, Chengdu Sport University, Chengdu, China; 3 School of Sports Training, Chengdu Sport University, Chengdu, China

**Keywords:** Plyometric training, Sports performance, Adolescents, Sprinting, Jumping, Agility

## Abstract

**Background:**

Plyometric training (PT) is a popular method of improving explosive power, speed, and agility in adolescent athletes. Nevertheless, earlier reviews have been mainly based on individual performance domains, individual sports or general youth groups. It is therefore not clear whether the effects of PT differ based on sex, type of sport, frequency of training or duration of intervention. This meta-analysis and systematic review investigated the impact of PT on jumping, sprinting, and agility performance in adolescents.

**Methods:**

PubMed, Web of Science, and Embase were searched from database inception to 1 January 2025, with an updated search on 30 January 2026. Randomized controlled trials that included healthy adolescents aged 10–19 years were included. Conventional random-effects models were used to pool the effects of PT on jumping, sprinting, and agility performance as standardized mean differences (SMDs) with 95% confidence intervals (CIs). To account for multiple effect sizes contributed by the same study, dependency-adjusted analyses were additionally conducted using three-level random-effects models with CR2 cluster-robust variance estimation.

**Results:**

A total of 63 studies were included. Conventional random-effects analyses showed that PT significantly improved jump performance (SMD = 0.648, 95% CI [0.523–0.772]; *P* < 0.001), sprint performance (SMD = −0.496, 95% CI [−0.609 to −0.383]; *P* < 0.001), and agility performance (SMD = −0.659, 95% CI [−0.851 to −0.467]; *P* < 0.001). Dependency-adjusted analyses confirmed the robustness of these findings for jump performance (SMD = 0.613, 95% CI [0.447–0.779]; *P* < 0.001), sprint performance (SMD = −0.414, 95% CI [−0.583 to −0.245]; *P* < 0.001), and agility performance (SMD = −0.595, 95% CI [−0.796 to −0.394]; *P* < 0.001). Benefits were more consistent with longer intervention durations, whereas sex- and sport-specific subgroup findings remained exploratory because of small subgroup sizes and heterogeneity.

**Conclusion:**

PT seems to be effective in enhancing jumping, sprinting, and agility performance among adolescents, especially when the interventions are continued at least 10 weeks. However, between-study heterogeneity, testing protocol variability, and small sample sizes in some sex- and sport-specific studies limit the generalizability of subgroup results. Future randomized controlled trials should use standardized outcome measures, report training dose and adherence in detail, and have a higher proportion of female participants. PROSPERO registration: CRD42024627316.

## Introduction

Adolescence is a critical period for motor skill development and refinement. The physical abilities gained at this age not only affect the short-term physical and psychological health but also the future sporting performance and lifetime engagement in physical exercise. Despite its importance, a worldwide decline in physical fitness among adolescents has been extensively documented. The World Health Organization states that over 80 percent of adolescents aged 11–17 years fail to comply with the recommended physical activity (Guthold et al., 2020). Moreover, there is evidence that a lack of motor skill development in the early years of life significantly decreases the chances of engaging in organized sports in adulthood, which is why early intervention is crucial (Söderström et al., 2018). Thus, the creation of scientifically based and effective training programs has significant clinical and population health implications to enhance athletic performance among adolescents.

Plyometric training (PT) has gained significant popularity in recent years due to its capacity to increase explosive power in a comparatively short time. PT is founded on the stretch-shortening cycle (Bchini et al., 2023) and is designed to enhance fast eccentric-concentric muscle movements that help to generate lower-limb power and explosive performance (Roberts, Nuckols & Krieger, 2020). It has been demonstrated that PT can significantly enhance the performance of adolescents in jumping, sprinting, and agility (Guadalupe-Grau et al., 2009; Moran et al., 2018; Ramirez-Campillo et al., 2020c). Nevertheless, the available data show that training results are quite variable, and biological maturity (*e.g.*, peak height velocity (PHV) status and timing) and sex are found to be significant moderators (Guadalupe-Grau et al., 2009; Moran et al., 2018). Past meta-analyses have indicated that the impacts of PT vary at the pre-, mid-, and post-PHV stages, and that there are sensitive periods during which certain motor skills are developed. As an illustration, male adolescents seem to show more significant changes in vertical jump performance in the pre- and post-PHV phases, but change-of-direction (COD) ability shows especially significant training effects in the PHV period (effect size (d) = 0.95–0.99) (Wang et al., 2024; Zheng et al., 2025). It is important to note that recent research has been mostly conducted in male adolescents, and the impact of PT in female adolescents is relatively under-researched (Oliver et al., 2024). Therefore, it is not clear how much sex differences affect PT outcomes. These results indicate that male and female adolescents may respond differently to PT, which can be explained by the fact that they are not yet physiologically developed and they are engaged in different sports activities.

The implications of PT on the performance of adolescent athletes are highly dependent on the performance measures that are employed to assess the performance. Jumping, sprinting, and agility are commonly known to be the important predictors of athletic performance among adolescents (Zheng et al., 2025). Jumping performance is mainly a measure of lower-limb explosive strength and neuromuscular coordination, sprinting performance is a measure of linear acceleration and maximal running speed, and agility performance is a measure of rapid directional changes and dynamic balance skills (Bariya, Patel & Pathak, 2019; Dardouri et al., 2023; Faamoe, 2024). These physical abilities should be enhanced to improve the overall athletic performance among adolescents. Since various athletic skills might result in different adaptive responses to PT, it is necessary to use proper and independent measures of assessment to determine the training outcomes accurately.

Despite the positive impact of PT in young athletes being proven by previous systematic reviews (Ramirez-Campillo et al., 2022; Sánchez et al., 2020) and meta-analyses (Oliver et al., 2024), there are still several significant gaps. Most of the reviews that have been done have been on one area of performance, particular sports or general youth groups as opposed to adolescents. Moreover, the degree to which PT effects depend on sex, sport discipline, training frequency per week, and intervention period has not been systematically studied. Moreover, the confidence of the evidence at hand and the risk of bias at the study level should be further synthesized. Thus, the purpose of this systematic review and meta-analysis of randomized controlled trials was to measure the impact of PT on jumping, sprinting, and agility performance in adolescents aged 10-19 years. Another aim was to investigate how these effects were moderated by intervention duration, training frequency per week, sex, and sport type. We theorized that PT would enhance all three performance outcomes. Nevertheless, the results of moderator analyses were deemed as exploratory since some of the subgroup categories had a small number of effect sizes.

## Survey Methodology

### Study registration

The research was carried out in line with the Preferred Reporting Items of Systematic Reviews and Meta-Analyses (PRISMA) guidelines (Page et al., 2021) and was pre-registered in the PROSPERO database (CRD42024627316).

### Data sources and search strategy

We used PubMed, Web of Science, and Embase databases since their inception until 1 January 2025. A new search was done on 30 January 2026, and no new eligible studies were found. The search strategy involved the use of controlled vocabulary terms, such as Medical Subject Headings (MeSH) where necessary, and free-text keywords with Boolean operators. Search strategies of each database, search fields, operators, and search dates are given in full in File S1.

### Inclusion and exclusion criteria

This review followed the PICOS framework to specify the inclusion criteria (Liberati et al., 2009). The sample was restricted to healthy adolescents between 10 and 19 years old (World Health Organization, 2018), and there were no limitations on sex, athletic performance, and training experience, which increases the applicability of the results. Past experience indicates that six-week-long training programs are enough to cause physiological changes and enhance athletic performance (Asadi et al., 2016; Slimani et al., 2016). Based on this, the following criteria were used to select eligible studies: the PT intervention was at least six weeks long, and the study was a randomized controlled trial that used a control group to isolate the effects of the intervention. Moreover, to guarantee methodological rigor and valid estimation of effects, studies had to report at least one quantifiable measure of athletic performance (*e.g.*, jump height, sprint speed, or agility) and present pre- and post-intervention group means and standard deviations (SDs). In order to preserve the integrity of the review, studies were excluded when they: included participants with medical or health conditions; did not include an element of PT; had an intervention duration of less than six weeks; included control groups that received other training interventions; did not provide the necessary outcome data; or were not randomized controlled trials.

### Study selection and data extraction

All the studies retrieved were imported into EndNote X9 to be managed centrally. Automated detection and manual verification were used to eliminate duplicate records. In order to have a thorough search, Wang and Lu also performed backward reference screening of the studies that were included in the review to determine whether there were any potentially relevant articles that were not identified in the process of the database search. Two reviewers, Cui and Tan, independently selected the studies based on a predesigned standardized data extraction form. The first screening was done by looking at titles and abstracts, and then full-text articles were obtained to undergo final eligibility evaluation. Data were extracted in terms of study characteristics, participant demographics, intervention details, and key performance outcomes. The main outcomes measured included jumping performance (countermovement jump, squat jump, and standing long jump), sprinting performance (10 m, 20 m, and 30 m sprint tests), and agility performance (*T*-test and COD ability). The same two reviewers independently extracted the data and then cross-checked it to ensure accuracy. Any disputes were sorted out by discussing with a third researcher (Chai) until an agreement was achieved. The outcome data extracted were group means and SDs of the data before and after the intervention. In the case of studies that presented data in a graphical form only, WebPlotDigitizer software was employed to extract the data digitally. In cases where data were missing or raw, the respective authors were approached to provide the same.

### Risk of bias and quality of evidence

Two reviewers independently evaluated risk of bias using Cochrane Risk of Bias 2 (RoB 2) tool in five domains: the randomization process, non-adherence to the intended interventions, missing outcome data, outcome measurement, and the choice of the reported result. In the domain of randomization process, the studies were considered to be of low risk only in case both random sequence generation and allocation concealment were described. The studies that indicated random assignment but lacked adequate methodological description were rated as having some concerns. In case of deviations of the intended interventions, judgments were made based on participant adherence, co-interventions, and possible contamination of the study groups. Any dispute between reviewers was settled by discussion and where no agreement was possible, a third reviewer gave the final adjudication.

### Certainty of evidence

The confidence of each of the main outcomes was determined by the Grading of Recommendations, Assessment, Development and Evaluations (GRADE) method, which takes into account the risk of bias, inconsistency, indirectness, imprecision, and publication bias. Detailed GRADE evaluations and justification of any downgrading judgments are presented in File S2.

### Statistical analysis

R statistical software and Stata 18 were used to perform meta-analyses, meta-regression analyses, and graphical outputs. The priority was given to change-from-baseline data to estimate the effects of interventions because the imbalance of the baselines is likely in participants aged 10–19 years and can bias the comparison of the post-intervention data. Random-effects models were used to express effect sizes in terms of SMDs with 95% CIs. The I^2^ statistic and tau^2^ were used to measure between-study heterogeneity.

Because some studies contributed more than one eligible effect size within the same outcome domain, we conducted additional analyses to address potential within-study dependence. First, one-effect-size-per-study sensitivity analyses were performed for jump, sprint, and agility performance using prespecified outcome hierarchies. For jump performance, the hierarchy was countermovement jump, followed by squat jump and standing long jump. For sprint performance, the hierarchy was 10 m, followed by 20 m and 30 m sprint tests. For agility performance, the hierarchy was the 505 COD test, followed by the *T*-test and COD speed tests. Second, to statistically account for non-independence among multiple effect sizes from the same study, dependency-adjusted analyses were conducted using three-level random-effects meta-analytic models. Effect sizes were nested within study identifiers, with random effects specified at the study and effect-size levels. Hedges’ g and its sampling variance were used as the effect-size estimate and corresponding variance. Cluster-robust variance estimation with CR2 adjustment and Satterthwaite degrees of freedom was applied using the clubSandwich package. These analyses were conducted in R 4.5.2 using the metafor and clubSandwich packages.

Subgroup analyses and meta-regression analyses were conducted to explore whether sex, sport type, weekly training frequency, and intervention duration were associated with the magnitude of training effects. Conventional random-effects models were first applied for comparability with previous meta-analyses. Dependency-adjusted subgroup and moderator analyses were then conducted using the same three-level framework described above. Moderators were chosen a priori according to training theory and available evidence: intervention duration and weekly frequency represented training dose, whereas sex and sport type represented participant characteristics and sport context. Analyses involving small subgroup sizes were considered exploratory.

The overall outcome level was used to assess publication bias with funnel plots and Egger’s test, and only when 10 or more independent studies were available on a particular outcome (Hayashino, Noguchi & Fukui, 2005). Subgroup analyses that had less than 10 effect sizes were not subjected to the Egger’s test because it is less powerful and unstable with small samples. Trim-and-fill analyses were conducted when funnel plot asymmetry or statistical evidence of small-study effects were identified to investigate the possible effect on pooled effect estimates (Berkey et al., 1995; Tantry et al., 2021).

The sensitivity analyses were done through leave-one-out procedures and by omitting studies with high risk of bias based on RoB 2. The studies that were rated as having some concerns were retained to maintain the available evidence base and evaluate the strength of the findings to the trials that were of higher risk.

Effect sizes were interpreted according to Cohen’s criteria: small (SMD < 0.2), moderate (0.2 ≤ SMD < 0.5), large (0.5 ≤ SMD < 0.8), and very large (SMD ≥ 0.8). Heterogeneity was classified as low (I^2^ < 25%), moderate (25% ≤ I^2^ < 75%), or high (I^2^ ≥ 75%) (Higgins & Thompson, 2002). Statistical significance was set at *P* < 0.05, and *P* values between 0.05 and 0.10 were interpreted only as possible trends. Because these moderator analyses were based on study-level associations, they were not interpreted as evidence of causality.

### Language restriction

No language restrictions were applied during study identification and screening.

## Results

### Study selection

**Figure 1:**
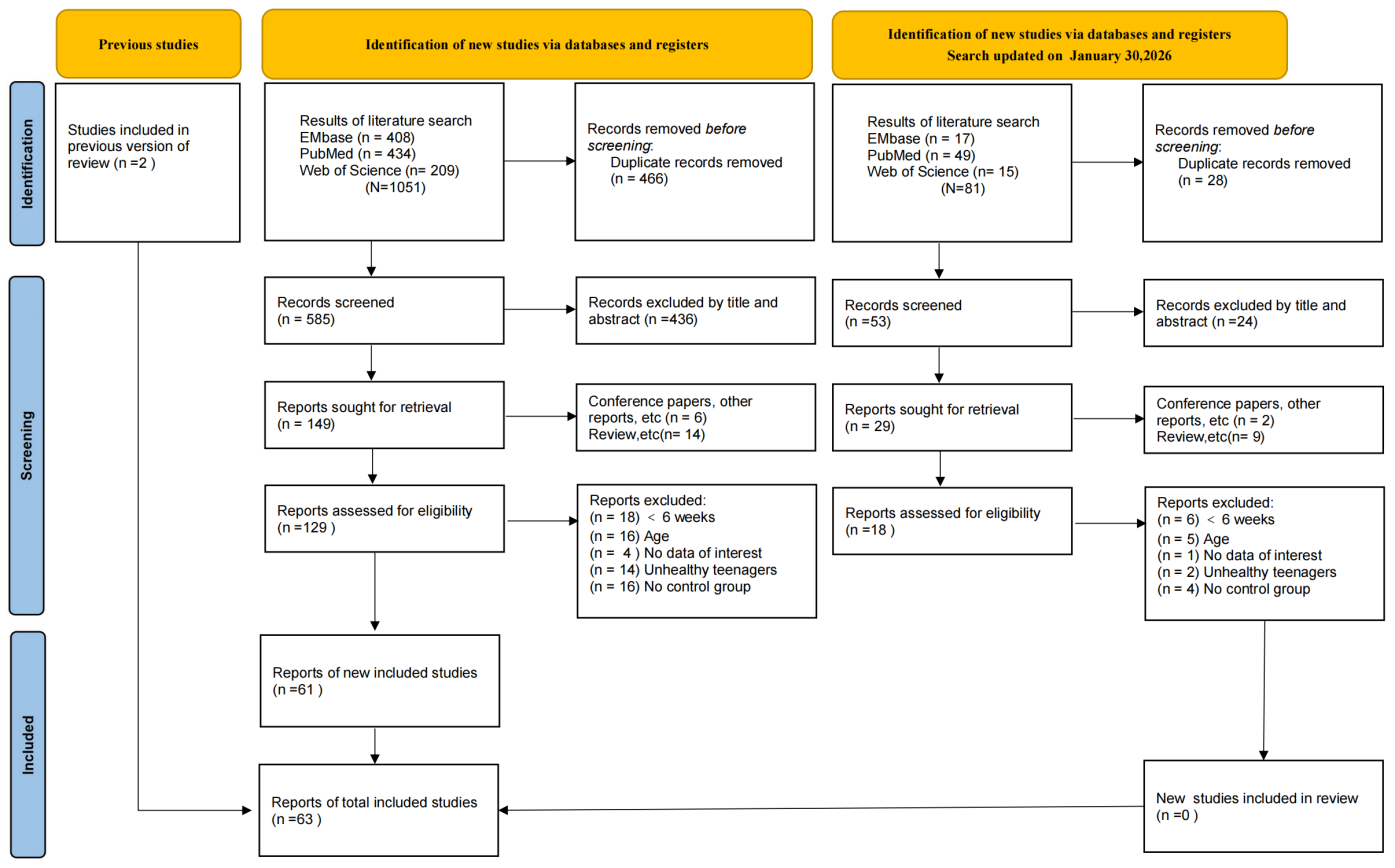
PRISMA flow diagram of the study selection.

The PRISMA guidelines suggest that an extensive literature search was performed in the Web of Science, PubMed, and Embase databases. One thousand one hundred and thirty-two records were retrieved, 1,051 of them were found during the first search, and 81 during the second search. Following the deletion of 466 duplicate records, 585 studies were left to undergo title screening, with 436 studies being eliminated because they were not related to the topic. The rest of the 149 articles were screened in terms of abstracts, and 20 articles were eliminated. Then, 129 full-text articles were evaluated on the basis of eligibility, and 68 were excluded because of the lack of intervention duration, improper age group, missing data, health status of the participants, or lack of a control group. The new search provided 81 more records, which were all excluded after full-text evaluation since they failed to meet the inclusion criteria. Moreover, the reference lists of the relevant review articles were screened, and two more eligible studies were identified. Finally, this systematic review and meta-analysis included 63 studies (Fig. 1).

### Study characteristics

The key features of the included studies are summarised in Table 1. Overall, 265 intervention and control groups were identified in the eligible studies. The participants were between 10 and 19 years old and were both male and female adolescents. The sports that were mostly used in the studies included soccer, basketball, volleyball, hockey, swimming, handball, and tennis. Ground-based PT was the main intervention programme and intervention periods were between 6 and 26 weeks. The frequency of training was 2–5 sessions per week, and the duration of the sessions was between 30 and 120 min. In total, 63 studies provided jumping results, 52 sprint results, and 23 agility results. Table 1 and the forest plots summarise the characteristics of the studies and the outcome allocations.

**Table 1:** Characteristics of studies included in this meta-analysis.

Author, year	Country	Age	Sex	Sport	N Exp	N Con	TP	TF	Main results
Aloui et al. (2021)	Tunisia	EG:14.6 ± 0.44	M	Soccer	17	16	8	2	①②
		CG:14.6 ± 0.39							
Aloui et al. (2022)	Tunisia	EG:14.6 ± 0.5	M	Soccer	17	17	8	5	①②
		CG:14.6 ± 0.4							
Attene et al. (2015)	Italy	EG:14.83 ± 0.92	F	Basketball	18	18	6	2	①
		CG:15.20 ± 0.92							
Beato et al. (2018)	Switzerland	EG: 17 ± 0.8	M	Soccer	11	10	6	2	①②③
		CG:17 ± 1.0	M						
Benito-MartíNez et al. (2013)	Spain	EG:17.05 ± 1.45	M and F	NA	19	20	8	2	①②
		CG:16.8 ± 1.54							
Bianchi et al. (2019)	Switzerland	17 ± 0.8	M	Soccer	10	11	8	4	①②③
Bouguezzi et al. (2020)	Tunisia	EG:11.32 ± 0.27	M	Soccer	15	15	8	1	①②
		CG:12.27 ± 0.33							
Bouteraa et al. (2020)	Tunisia	EG:16.4 ± 0.5	F	Basketball	16	10	8	2	①②
		CG:16.5 ± 0.5							
Buğa & Gencer (2022)	Türkiye	EG:13.17 ± 1.11	M	Basketball	12	12	8	2	①②③
		CG:13.58 ±.90							
Chaabene & Negra (2017)	Tunisia	EG:12.68 ± 0.23	M	Soccer	13	12	8	2	①②
		CG:12.72 ± 0.27							
Chaabene et al. (2019)	Germany	EG:15.9 ± 0.2	F	Handball	12	9	8	2	①②
		CG:15.9 ± 0.3							
Chaouachi et al. (2017)	Tunisia	EG:13.9 ± 0.27	M	Soccer	13	13	8	4	①②
		CG:13.8 ± 0.32							
Chelly et al. (2014)	Tunisia	EG:17.1 ± 0.3	M	Handball	12	11	8	2	①②
		CG:17.1 ± 0.4							
Chtara et al. (2017)	Tunisia	13.6 ± 0.3	M	Soccer	10	10	6	2	①②③
Davies et al. (2021)	United Kingdom	EG:11.7 ± 0.6	F	NA	33	40	7	2	①②
		CG:11.3 ± 0.7							
Falch et al. (2022)	Norway	EG:17.1 ± 2.4	F	Handball	10	11	6	2	①②
		CG:17.5 ± 2.3							
Fathi et al. (2019)	Tunisia	EG:14.6 ± 0.5	M	volleyball	20	20	16	2	①②
		CG:14.5 ± 0.6							
Ferley, Scholten & Vukovich (2020)	United States	EG:15.71 ± 1.28	M and F	Soccer	17	15	8	3	①②③
		CG:16.04 ± 1.21	M and F						
Fernandez-Fernandez et al. (2016)	Spain	EG: 12.5 ± 0.3	M	Tennis	24	27	8	3	①②③
		CG:12.5 ± 0.3							
Franco-Márquez et al. (2015)	Spain	14.7 ± 0.5	M	Soccer	18	20	6	2	①
Gaamouri et al. (2023)	Tunisia	15.8 ± 0.2	F	Handball	14	14	10	2	①
Gonzalo-Skok et al. (2019)	Spain	13.3 ± 0.7	M	Basketball	9	9	6	2	①②
Hammami et al. (2020c)	Tunisia	15.8 ± 0.2	F	Handball	17	17	10	2	①②
Hammami et al. (2020a)	Qatar	EG:16.4 ± 0.5	M	Handball	10	10	7	3	①②③
		CG:16.5 ± 0.4							
Hammami et al. (2020b)	Tunisia	EG:16.2 ± 0.2	M	Soccer	12	12	10	2	①②
		CG:16.4 ± 0.2							
Hammami et al. (2019)	Tunisia	EG:15.7 ± 0.2	M	Soccer	14	12	8	2	①②
		CG:15.8 ± 0.2							
Hammami et al. (2021)	Tunisia	EG:16.6 ± 0.5	M	Handball	17	15	8	2	①②③
		CG:16.5 ± 0.8							
Hernandez-Martinez et al. (2023)	Chile	EGI:14.6 ± 0.89	M	Volleyball	EGI:15	12	8	2	①②
		EGII:14.5 ± 0.57			EGII:14				
		CG:15.0 ± 0.85							
De Hoyo et al. (2016)	Spain	18 ± 1	M	Soccer	9	11	8	2	①②
Idrizovic et al. (2017)	Kosovo	EGI:16.6 ± 0.6	F	Volleyball	EGI:13	17	12	2	①②
		EGII:16.6 ± 0.6			EGII:17				
		CG:16.6 ± 0.6							
Jlid et al. (2019)	Tunisia	EG:11.8 ± 0.4	M	Soccer	14	14	8	2	①③
		CG:11.6 ± 0.5							
Kobel et al. (2017)	Brazil	15.9 ± 1.2	M	Soccer	9	11	6	2	①②
Kryeziu et al. (2023)	North Macedonia	EG:15.9 ± 2.6	M	NA	90	105	12	3	①②
		CG:15.6 ± 2.3							
Kurt et al. (2023)	Türkiye	12.09 ± 0.89	M	Soccer	11	11	6	2	①②③
Liu, Wang & Xu (2024)	Thailand	16.3 ± 0.6	M	Soccer	EGI:17	17	8	4	①②
					EGII:17				
Lloyd et al. (2016)	United Kingdom	EG:12.7 ± 0.3	M	NA	10	10	6	2	①②
		CG:12.8 ± 0.2							
Makhlouf et al. (2018)	Tunisia	EGI:11.06 ± 0.75	M	Soccer	EGI:21	16	8	2	①②
		EGII:11.29 ± 0.85			EGII:20				
		CG:10.98 ± 0.80							
Marzouki et al. (2023)	Tunisia	EG:16.3 ± 0.2	M	Soccer	12	12	8	2	①②③
		CG:16.2 ± 0.3							
McKinlay et al. (2018)	Canada	EGI:12.5 ± 0.7	M	Soccer	EGI:14	14	8	2	①
		EGII:12.6 ± 0.7			EGII:13				
		CG:12.5 ± 0.3							
Meszler & Váczi (2019)	Hungary	EG:15.8 ± 1.2	F	Basketball	9	9	7	2	①
		CG:15.7 ± 1.3							
Meylan & Malatesta (2009)	New Zealand	EG:13.3 ± 0.6	M	Soccer	14	11	8	2	①
		CG:13.1 ± 0.6							
Moran et al. (2016)	United Kingdom	EGI:12.6 ± 0.7	M	Hockey	EGI:9	CGI:12	6	2	①②
		CGI:12.8 ± 0.8			EGII:8	CGII:9			
		EGII:14.3 ± 0.6							
		CGII:14.4 ± 0.5							
Negra et al. (2017b)	Tunisia	EG:12.1 ± 0.5	M	Soccer	17	16	8	2	①
		CG:12.2 ± 0.6							
Negra et al. (2017a)	Tunisia	EG:12.7 ± 0.2	M	Soccer	18	14	8	2	①②
		CG:12.2 ± 0.5							
Negra et al. (2020)	Tunisia	12.7 ± 0.2	M	Soccer	13	11	8	2	①②
Norgeot & Fouré (2024)	France	14.5 ± 0.5	M	Soccer	14	14	8	2	①②
Novak et al. (2023)	Croatia	13.5 ± 1.8	M and F	Tennis	15	15	6	2	①②③
			M and F						
Padrón-Cabo et al. (2021)	Spain	EG:12.60 ± 0.70	M	Soccer	10	10	6	2	①②③
		CG:12.39 ± 0.56							
Palma-Muñoz et al. (2021)	Chile	EG:14.6 ± 1.1	M	Basketball	7	7	6	2	①②
		CG:14.0 ± 2.0							
Potdevin et al. (2011)	France	EG:14.3 ± 0.2	M and F	Swimming	12	11	6	2	①
		CG:14.1 ± 0.2	M and F						
Ramirez-Campillo et al. (2020b)	Iran	EG:17.1 ± 0.3	M	Soccer	12	14	7	2	①②③
		CG:17.1 ± 0.5							
Ramirez-Campillo et al. (2020a)	Chile	EG:12.1 ± 2.2	M	Soccer	8	7	8	2	①②③
		CG:12.6 ± 1.8							
Ramirez-Campillo et al. (2018)	Chile	EG:13.1 ± 1.7	M	Soccer	24	24	7	2	①②③
		CG:13.7 ± 1.6							
Ramirez-Campillo et al. (2019)	Chile	EG:13.2 ± 1.8	M	Soccer	19	20	7	2	①②③
		CG:13.5 ± 1.9							
Rubley et al. (2011)	United States	13.4 ± 0.5	F	Soccer	10	6	14	1	①
Sáez de Villarreal et al. (2021)	Spain	EG:13.57 ± 1.39	M	Basketball	10	10	7	2	①②③
		CG:14.66 ± 0.86							
Sáez de Villarreal et al. (2015)	Spain	EG:15.33 ± 0.34	NA	Soccer	13	13	26	2	①②③
		CG:14.90 ± 0.17							
Sammoud et al. (2024)	Tunisia	EG:12.7 ± 0.2	M	Soccer	13	14	8	5	①②③
		CG:11.8 ± 0.4							
Santos & Janeira (2011)	Portugal	EG:15.0 ± 0,5	M	basketball	14	10	10	2	①
		CG:14.5 ± 0.4							
Söhnlein, Müller & Stöggl (2014)	Ghana	EG:13.0 ± 0.9	M	Soccer	12	10	16	2	①②③
		CG:12.3 ± 0.8							
Söyler et al. (2024)	Türkiye	EG:16.71 ± 0.47	M	Soccer	14	14	8	5	①②
		CG:16.64 ± 0.50							
Thaqi, Berisha & Hoxha (2020)	Kosovo	16 ± 0.5	M	NA	110	110	12	3	①②
Vera-Assaoka et al. (2020)	Chile	EGI:11.2 ± 0.8	M	Soccer	EGI:16	CGI:16	7	2	①②③
		EGII:14.4 ± 1.0			EGII:22	CGII:22			
		CGI:11.5 ± 0.9							
		CGII:14.5 ± 1.1							

**Notes.**

EG experimental group G control group F female M male ① jumping ② sprinting ③ agility NA not report TP intervention period TF intervention frequency

### Risk of bias in studies

**Figure 2:**
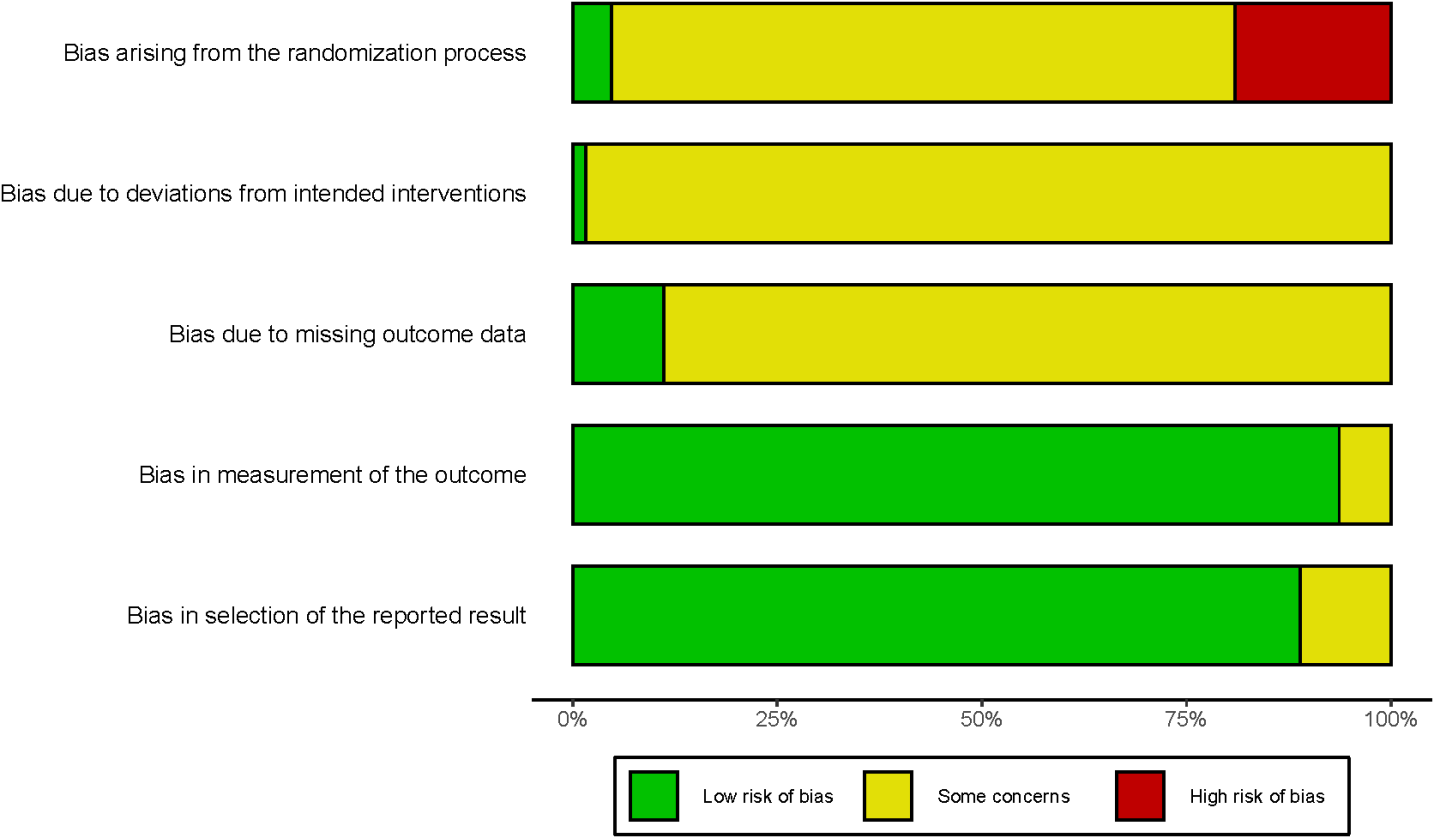
Risk of bias graph.

The Cochrane RoB 2 tool was used to evaluate the risk of bias. In the studies included, the most common areas of concern were the randomization process and non-adherence to the intended interventions, but the outcome measurement was typically considered to be at low risk of bias. Overall risk-of-bias judgments (low risk, some concerns, and high risk) are presented in Fig. 2.

### Publication bias

**Figure 3:**
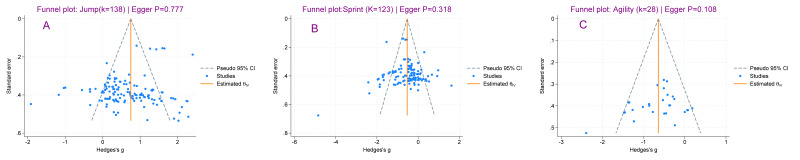
Publication bias. (A) Jump (B) Sprint (C) Agility.

On the overall outcome level, the visual inspection of the funnel plots did not show significant asymmetry. Moreover, Egger test did not show statistically significant evidence of publication bias in jumping (*P* = 0.777), sprinting (*P* = 0.318), or agility (*P* = 0.108) results (Fig. 3).

### Meta-analysis of PT on adolescent sports performance

#### Effects of PT on jumping performance

Sixty-three studies provided 138 effect sizes of jump performance. The heterogeneity was high (I^2^ = 75.4%), and thus random-effects models were used. PT was associated with improved jump performance (SMD = 0.648, 95% CI [0.523–0.772]; *P* < 0.001) (Table 2). In the dependency-adjusted three-level model with CR2 robust variance estimation, the effect remained statistically significant (SMD = 0.613, 95% CI [0.447–0.779]; *P* < 0.001). These estimates should be interpreted as average effects across heterogeneous populations, testing procedures, and intervention designs.

**Table 2:** Effects of plyometric training on jump performance (overall and subgroup analyses).

Analysis/Subgroup	k	SMD (Hedges’g)	95% CI	*P*	I^2^ (%)
Overall	138	0.648	0.523 to 0.772	<0.001	75.4
Sex					
M (Male)	110	0.616	0.472 to 0.759	<0.001	77.6
F (Female)	19	0.856	0.525 to 1.187	<0.001	70.4
Sport					
Soccer	82	0.455	0.314 to 0.596	<0.001	62.3
Handball	14	1.472	1.108 to 1.836	<0.001	58.1
Basketball	14	0.570	0.346 to 0.795	<0.001	0.0
Volleyball	11	0.719	0.424 to 1.013	<0.001	42.1
Duration					
6–7 weeks	37	0.398	0.269 to 0.527	<0.001	0.0
8 weeks	75	0.521	0.366 to 0.677	<0.001	67.1
10–26 weeks	26	1.278	1.029 to 1.528	<0.001	79.1

**Notes.**

k, effect sizes.

Table 2 shows subgroup analyses of jump performance. Both sex subgroups showed improvements, but the female subgroup had fewer effect sizes and thus less accurate estimates. The effects of sport-specific interventions were different in different disciplines, but the duration of interventions had the most consistent pattern, with bigger effects in longer interventions. In general, these subgroup results can be viewed as exploratory, as the heterogeneity is high, and the subgroup sizes are unequal.

#### Effects of PT on sprint performance

A total of 52 studies with 123 effect sizes were included in Sprint performance analyses. The heterogeneity was moderate to high (I^2^ = 66.2%), and a random-effects model was thus used. PT was associated with improved sprint performance (SMD = −0.496, 95% CI [−0.609 to −0.383]; *P* < 0.001) (Table 3). This finding was confirmed in the dependency-adjusted three-level model with CR2 robust variance estimation (SMD = −0.414, 95% CI [−0.583 to −0.245]; *P* < 0.001).

**Table 3:** Effects of plyometric training on sprint performance (overall and subgroup analyses).

Analysis/Subgroup	k	SMD (Hedges’g)	95% CI	*P*	I^2^ (%)
Overall	123	−0.496	−0.609 to −0.383	<0.001	66.2
**Sex**					
M (Male)	99	−0.446	−0.565 to −0.328	<0.001	62.4
F (Female)	16	−0.892	−1.392 to −0.393	<0.001	83.8
**Sport**					
Soccer	69	−0.431	−0.576 to −0.286	<0.001	56.7
Handball	19	−1.041	−1.512 to −0.569	<0.001	82.0
Basketball	8	−0.280	−0.592 to 0.031	0.078	0.0
Volleyball	8	−0.232	−0.483 to 0.018	0.069	0.0
**Duration**					
6–7 weeks	41	−0.193	−0.318 to −0.069	0.002	0.0
8 weeks	55	−0.496	−0.674 to −0.318	<0.001	66.6
10–26 weeks	27	−0.907	−1.153 to −0.662	<0.001	78.0

**Notes.**

k, effect sizes.

Table 3 summarizes subgroup analyses of sprint performance. The overall effects of PT were positive in sex subgroups, but the female subgroup had fewer effect sizes and thus less accurate estimates. The sport-specific results were less consistent and some of the categories were backed by a small amount of evidence. The most consistent pattern was in intervention duration, where longer interventions had more improvements. Based on this, subgroup comparisons must be viewed with caution and not as conclusive evidence of different effects.

#### Effects of PT on agility performance

In the case of agility performance, 23 studies provided 28 effect sizes. Heterogeneity was low to moderate (I^2^ = 39.8%), and random-effects models were therefore used. In the conventional random-effects analysis, PT was associated with improved agility performance (SMD = −0.659, 95% CI [−0.851 to −0.467]; *P* < 0.001) (Table 4). The dependency-adjusted three-level model with CR2 robust variance estimation produced a consistent result (SMD = −0.595, 95% CI [−0.796 to −0.394]; *P* < 0.001).

**Table 4:** Effects of plyometric training on agility performance (overall and subgroup analyses).

Analysis/Subgroup	k	SMD (Hedges’g)	95% CI	*P*	I^2^ (%)
Overall	28	−0.659	−0.851 to −0.467	<0.001	39.8
Sport					
Soccer	22	−0.656	−0.887 to −0.426	<0.001	45.5
Handball	2	−1.302	−1.906 to −0.699	<0.001	0.0
Basketball	2	−0.431	−1.030 to 0.168	0.158	0.0
Tennis	2	−0.426	−0.867 to 0.015	0.058	0.0
Duration					
6–7 weeks	13	−0.499	−0.726 to −0.272	<0.001	14.7
8 weeks	10	−0.642	−1.010 to −0.274	<0.001	53.0
10–26 weeks	5	−1.193	−1.576 to −0.810	<0.001	0.0

**Notes.**

k, effect sizes.

Table 4 shows subgroup analyses of agility performance. Sport-specific estimates indicated that soccer and handball improved most, but a number of sport categories had very few effect sizes, which restricted the validity of between-sport comparisons. Time-based analyses showed that there was a possible trend of larger effects with longer interventions, but the subgroup with the longest duration had few studies. These subgroup results, therefore, are to be viewed as exploratory.

### Certainty of evidence

Based on the GRADE assessment, the certainty of evidence was rated as moderate for jump performance and low for both sprint and agility performance. The jump outcome was downgraded primarily due to serious inconsistency arising from substantial heterogeneity. The sprint outcome was downgraded due to concerns regarding risk of bias and inconsistency, whereas the agility outcome was downgraded for imprecision and indirectness, largely related to the smaller evidence base and variability in agility testing protocols. A concise Summary of Findings table is presented in Table 5, and detailed GRADE judgments are provided in File S2.

**Table 5:** Summary of findings and certainty of evidence assessed using GRADE.

Outcome	Studies/ effect sizes	Participants	Pooled effect, SMD, 95% CI	I^2^	Certainty of evidence	Main reasons for downgrading
Jump	63 / 138	4,904	0.648, 0.523 to 0.772	75.40%	Moderate	Downgraded one level for serious inconsistency due to substantial heterogeneity.
Sprint	51/123	4,316	−0.496, −0.609 to −0.383	66.20%	Low	Downgraded for risk-of-bias concerns and inconsistency due to moderate-to-substantial heterogeneity.
Agility	22 / 28	746	−0.659, −0.851 to −0.467	39.80%	Low	Downgraded for imprecision and indirectness due to the smaller evidence base and variation in agility testing protocols.

### Dependency-adjusted duration

Dependency-adjusted duration-based analyses showed a consistent pattern of larger effects in longer interventions. For jump performance, the estimated effects were SMD = 0.427 (95% CI [0.241–0.613]) for 6–7 weeks, SMD = 0.507 (95% CI [0.259–0.755]) for 8 weeks, and SMD = 1.243 (95% CI [0.818–1.668]) for 10–26 weeks. For sprint performance, the corresponding estimates were SMD = −0.194 (95% CI [−0.347 to −0.040]), SMD = −0.419 (95% CI [−0.704 to −0.133]), and SMD = −0.824 (95% CI [−1.314 to −0.334]). For agility performance, the estimates were SMD = −0.487 (95% CI [−0.751 to −0.222]), SMD = −0.612 (95% CI [−0.952 to −0.273]), and SMD = −1.170 (95% CI [−4.005 to 1.665]), respectively. The longest-duration agility subgroup should be interpreted cautiously because it included only two study clusters.

### Meta-regression analysis

Table 6 presents the dependency-adjusted moderator analyses based on three-level meta-regression models with CR2 robust variance estimation. Intervention duration remained the most consistent moderator. Compared with the 6-7 week category, the 10-26 week category was associated with larger improvements in jump performance (difference = 0.816, 95% CI [0.379–1.253]; *P* < 0.001) and sprint performance (difference = −0.630, 95% CI [−1.104 to −0.156]; *P* = 0.012). The corresponding difference for agility performance was not statistically significant after dependency adjustment (difference = −0.683, 95% CI [−2.100 to 0.734]; *P* = 0.145) and should be interpreted cautiously because the longest-duration agility subgroup included only five effect sizes from two study clusters. Weekly training frequency and sex did not show consistent moderator patterns across outcomes. Sport-type analyses were considered exploratory because several categories contained small numbers of study clusters. These moderator findings should be interpreted as exploratory study-level associations rather than causal effects.

**Table 6:** Dependency-adjusted meta-regression analyses exploring heterogeneity.

**Outcome**	**Moderator**	**Comparison**	**β/ Difference in SMD**	**95% CI**	***P* value**	**Effect sizes**	**Study clusters**
Jump	Continuous duration	Per additional intervention week	0.052	−0.041 to 0.145	0.176	138	63
Jump	Weekly frequency	Per additional session/week	−0.052	−0.338 to 0.234	0.684	138	63
Jump	Duration category	8 weeks *vs* 6–7 weeks	0.080	−0.224 to 0.384	0.597	138	63
Jump	Duration category	10–26 weeks *vs* 6–7 weeks	0.816	0.379 to 1.253	<0.001	138	63
Jump	Sex	Female *vs* male	0.083	−0.413 to 0.579	0.725	136	62
Sprint	Continuous duration	Per additional intervention week	−0.042	−0.092 to 0.009	0.080	123	52
Sprint	Weekly frequency	Per additional session/week	−0.066	−0.305 to 0.172	0.526	123	52
Sprint	Duration category	8 weeks *vs* 6–7 weeks	−0.225	−0.541 to 0.091	0.157	123	52
Sprint	Duration category	10–26 weeks *vs* 6–7 weeks	−0.630	−1.104 to −0.156	0.012	123	52
Sprint	Sex	Female *vs* male	−0.326	−1.111 to 0.458	0.366	121	51
Agility	Continuous duration	Per additional intervention week	−0.041	−0.085 to 0.002	0.054	28	23
Agility	Weekly frequency	Per additional session/week	0.056	−0.274 to 0.386	0.614	28	23
Agility	Duration category	8 weeks *vs* 6–7 weeks	−0.126	−0.523 to 0.272	0.512	28	23
Agility	Duration category	10–26 weeks *vs* 6–7 weeks	−0.683	−2.100 to 0.734	0.145	28	23

**Notes.**

Values are from three-level random-effects meta-regression models with effect sizes nested within study clusters. CR2 cluster-robust variance estimation with Satterthwaite degrees of freedom was used. *β* represents the difference in SMD relative to the reference category or the change in SMD per one-unit increase in the continuous moderator. For sprint and agility outcomes, negative values indicate improved performance when lower test times represent better performance. Sport-type moderator coefficients are provided in Supplementary File 4 because several sport-specific categories contained small numbers of study clusters. Study clusters refer to unique study-level identifiers used in the dependency-adjusted analyses. Multiple effect sizes contributed by the same study cluster were modelled as dependent within the three-level framework. Cluster counts may vary across moderators because not all studies contributed data to each analysis.

### Sensitivity analysis

Sensitivity analyses showed that the omission of single studies did not significantly change the direction of the pooled effects. Following the elimination of studies with a high risk of bias, both jump and sprint performance pooled estimates were consistent in direction, but heterogeneity was reduced. For jump performance, I^2^ decreased from 75.4% to 48.0%, and tau^2^ decreased from 0.39 to 0.14. For sprint performance, I^2^ decreased from 66.2% to 41.0%, and tau^2^ decreased from 0.24 to 0.11. None of the studies that contributed to the agility result were considered to be of high risk of bias, thus, the pooled effect and heterogeneity estimates of agility did not change (I^2^ = 39.8%, tau^2^ = 0.10). These results indicate that high-risk studies added to the heterogeneity in the jump and sprint analyses, but did not significantly affect the direction of the pooled effects.

One-effect-size-per-study sensitivity analyses were retained as an additional robustness check. The pooled effects for jump, sprint, and agility performance remained statistically significant and were consistent in direction with both the conventional random-effects analyses and the dependency-adjusted three-level analyses. Detailed one-effect-size-per-study sensitivity analyses are presented in File S3, and the dependency-adjusted model outputs, including the sport-type moderator analyses, are presented in File S4.

## Discussion

The findings of this systematic review and meta-analysis suggest that PT is associated with improved jumping, sprinting, and agility performance in adolescents. These findings remained statistically significant after dependency-adjusted three-level modelling with CR2 robust variance estimation, indicating that the main conclusions were not driven solely by multiple effect sizes from the same studies. Nevertheless, heterogeneity was high for jumping and moderate to high for sprinting, and the pooled estimates should be interpreted as average effects across heterogeneous populations, sports, testing protocols, and intervention designs rather than as uniform effects applicable to all adolescent athletes. Intervention duration was the most consistent study-level moderator, whereas sex- and sport-specific findings were less stable and should be viewed as exploratory.

### Effects of PT on jumping performance in adolescents

This meta-analysis, which combines 138 effect sizes, suggests that PT is linked with better jump performance in adolescents (SMD = 0.648, *P* < 0.001). This result aligns with the prior studies that have found positive impacts of PT on lower-limb power-related outcomes (Ramírez-dela Cruz et al., 2022). Notably, unlike most of the available literature, which has concentrated on PT in injury rehabilitation or clinical groups (Buckthorpe & Della Villa, 2021), the current study goes a step further to quantify its ergogenic effects in healthy adolescents, a critical group in long-term athletic development. The size of the effect here (SMD = 0.648) is within the scope of previous estimates by Markovic (2007) who found effect sizes of 0.44 to 0.88 with different PT protocols. The existing findings, based on a significantly larger evidence base, are generally consistent with those findings, but the heterogeneity is quite high, implying that the effects might be different in different populations, training regimens, and testing conditions. These performance improvements can be in line with neuromuscular factors such as improved stretch-shortening cycle activity and better eccentric control. Nonetheless, since the majority of the included trials did not directly evaluate tendon stiffness, neural drive, or muscle–tendon unit adaptations, these mechanisms can be viewed as hypothetical explanations instead of empirically validated pathways in this meta-analysis. These hypotheses can be further contextualized by biomechanical and computational modelling studies. As an example, Mănescu (2025) compared training tasks with OpenSim musculoskeletal simulations and found that plyometric movements produced higher ground reaction forces, higher early rate of force development (RFD) and higher activation synchrony than traditional resistance training tasks. Specifically, peak ankle joint moment, early RFD during 0-100 ms, and activation synchrony were found to be the most important predictors of performance-related outputs. Nevertheless, the results of these modelling studies cannot be viewed as direct evidence that neural drive or muscle–tendon adaptations mediated the effects of the included trials (Mănescu, 2025).

In sex-stratified analyses, both male and female adolescents seemed to be advantaged by PT, but the female estimate was calculated on fewer effect sizes and thus should be viewed with less accuracy. The evidence of sex-specific adaptations to PT is inconclusive, and the numerically larger effect in females could be due to differences in baseline fitness, maturational status, or previous training exposure, and not a sex-specific response. Future experiments ought to involve more female samples and report more on maturation status, baseline performance, and training history.

There were sport-specific estimates of jump performance, which differed among disciplines, but these differences should not be construed as a clear ranking of sports. The seemingly greater effect in handball could be due to variations in baseline fitness levels, intervention design, outcome selection or other study-level factors and not a sport-specific biological reaction to PT. In sports like volleyball, basketball, and soccer, athletes usually engage in regular training and competition that involves frequent plyometric-like movements, which can potentially nullify the relative added value of structured PT. In general, sport-specific results should be viewed as hypothesis-generating and need to be validated in research with similar protocols and sufficiently powered sport-specific samples.

Duration-based comparisons, including the dependency-adjusted analyses, indicated that longer PT programs were associated with larger average improvements in jump performance. This pattern may reflect greater cumulative training exposure, progressive overload, or differences in training volume and participant characteristics. However, these explanations remain hypothetical because most included studies did not directly assess neural adaptations, tendon morphology or stiffness, muscle morphology, or muscle–tendon unit adaptations. Therefore, duration-related findings should be interpreted as exploratory study-level associations rather than proof that longer PT interventions causally produce superior jump adaptations in all adolescents.

### Effects of PT on sprint performance in adolescents

This review indicates that PT is linked to better sprint performance in adolescents, but moderate-to-substantial heterogeneity suggests that the effects differ across studies. The analysis by Wang et al. (2024) showed greater effects of sprints, but comparisons with the current review should be approached with caution due to the differences in the sports included, training regimens, and sprint outcome measures. Certain PT protocols can be more consistent with the acceleration requirements of short-distance sprint tests, although this description is speculative due to the inconsistencies in protocol design and outcome definitions across studies. The current meta-analysis has not directly evaluated eccentric control, isometric stability, maximal strength, or tendon stiffness; hence, these processes cannot be validated. Moreover, the variability in training responses can also be caused by inter-individual differences in neuromuscular development and hormonal status during adolescence (Almeida-Neto et al., 2020).

Sex-stratified sprint analyses showed positive results in both sexes; the female subgroup was smaller and less accurate. In line with this, the existing evidence does not indicate a conclusive finding that PT is more effective in females. Future studies ought to further investigate sex-specific responses with sufficiently powered female samples and report in detail maturation status, baseline sprint performance, and training exposure.

The sport-specific sprint results were not consistent and were to be viewed with care. The comparatively greater effect in handball could be due to the repeated acceleration-deceleration and COD requirements of the sport, but the small size of subgroups and the variability of intervention protocols limit definitive interpretation. Soccer also showed positive results, but the estimate of basketball was not accurate and cannot be taken as the sign of poor effectiveness. In general, the variation in sports could be explained by the level of baseline performance, maturational status, dose of training, distance of sprint test, and the design of the program.

Duration analyses, including the dependency-adjusted moderator models, indicated larger average sprint improvements with longer interventions. This pattern may reflect greater cumulative training exposure and more progressive overload, but the comparisons were indirect and intervention characteristics varied across studies. Importantly, these moderator results should not be interpreted as causal evidence. The present review cannot determine the physiological processes underlying the observed pattern because most included trials did not directly measure tendon properties, neural adaptations, muscle morphology, or other mechanistic outcomes (Harries et al., 2018; Slimani et al., 2016).

### Effects of PT on agility performance in adolescents

The findings indicate that PT may improve change-of-direction–related performance in adolescents. This is physically possible, because agility exercises generally involve quick deceleration, re-acceleration, and good control of lower-limb force. Agility is a performance determinant in most youth sports as well, and could be linked to injury risk (Anniza & Iskandar, 2022; Mackala et al., 2020; Paul, Gabbett & Nassis, 2016). However, the lack of consistency in agility tests and intervention regimes makes it difficult to compare studies directly, and personal training condition can also affect responsiveness.

The results of sport-specific agility should be viewed as exploratory because a number of categories were backed by a very small number of effect sizes. The apparent differences between soccer, handball, basketball, and tennis might be due to sport-specific movement requirements, the level of agility at the baseline, the design of training, and the type of agility tests; an example is that team sports vary in their requirements of high-speed running, braking, and COD movements (Nygaard Falch, Guldteig Rædergård & Van den Tillaar, 2019). The non-significant or imprecise estimates in tennis and basketball cannot be taken as the evidence of ineffectiveness due to the small evidence base. Standardized agility assessments should be used in larger sport-specific trials.

Duration-based agility analyses showed a possible pattern of larger effects with longer interventions, but this finding was less stable after dependency adjustment than the corresponding jump and sprint findings. In particular, the longest-duration agility subgroup included only five effect sizes from two study clusters, resulting in wide confidence intervals. Therefore, agility duration findings should be treated as hypothesis-generating and not prescriptive. Future trials should use standardized agility assessments and report sufficient mechanistic and training-dose information to clarify whether longer PT interventions produce distinct agility adaptations.

## Limitations

These findings have a number of limitations that should be taken into account. To begin with, the chosen outcomes, jumping, sprinting, and agility performance, are not all-encompassing of adolescent physical development. Future research must thus include other indicators like muscular strength, balance, and other neuromotor abilities to give a more detailed assessment of sports performance in adolescents. Second, not all of the studies included had large sample sizes, especially those that investigated the effects of PT in female adolescents, which could decrease the accuracy and strength of sex-specific estimates. Moreover, the available literature has been mostly on short-term interventions and few studies have been conducted on long-term training effects. The interplay of training cycles, frequency, and periodization should thus be studied in future research to gain a better insight into the long-term adaptive responses and to guide the development of sustainable training programs. Third, there was a high level of methodological heterogeneity among the studies, especially in sprint, jump, and agility tests. The application of various sprint distances, jump protocols, and agility tests probably led to residual heterogeneity and could restrict the direct comparability of pooled estimates. Other limitations are the lack of reporting of training dose and adherence, insufficient data on the biological maturation status of the participants, and small subgroups in sex- and sport-specific analyses. Finally, although we conducted dependency-adjusted three-level models with CR2 robust variance estimation to account for multiple effect sizes from the same study, several moderator analyses still involved small subgroup sizes and low effective degrees of freedom. This was particularly relevant for the longest-duration agility subgroup and some sex- and sport-specific categories. Therefore, subgroup and meta-regression findings should still be interpreted as exploratory study-level associations rather than causal or prescriptive evidence.

## Conclusions

PT appears to improve jumping, sprinting, and agility performance in adolescents, and these main findings remained robust after dependency-adjusted three-level modelling. Longer interventions showed the most consistent association with larger performance improvements, especially for jump and sprint outcomes. Nevertheless, subgroup and meta-regression findings should be interpreted as exploratory because of between-study heterogeneity, variability in testing protocols, and limited sample sizes or study clusters in some sex-, sport-, and duration-specific categories. Future randomized controlled trials should use standardized outcome measures, report training dose and adherence in detail, include a higher proportion of female participants, and directly assess mechanistic outcomes such as muscle morphology, tendon properties, and neural adaptations.

## Supplemental Information

10.7717/peerj.21585/supp-1 Supplemental Information 1 Search strategy

10.7717/peerj.21585/supp-2 Supplemental Information 2 GRADE assessment of evidence quality for outcomes

10.7717/peerj.21585/supp-3 Supplemental Information 3 One-effect-size-per-study sensitivity analyses

10.7717/peerj.21585/supp-4 Supplemental Information 4 Dependency-adjusted analyses

10.7717/peerj.21585/supp-5 Supplemental Information 5 R script and cleaned effect-size dataset

10.7717/peerj.21585/supp-6 Supplemental Information 6 Forest plot of standardized mean differences (Hedges g) for agility outcomes, with results organized by intervention duration and summarized using a random-effects model

10.7717/peerj.21585/supp-7 Supplemental Information 7 Forest plot showing the agility outcomes for studies with 6- to 7-week plyometric training interventions

10.7717/peerj.21585/supp-8 Supplemental Information 8 Forest plot showing the agility outcomes for studies with 8-week plyometric training interventions

10.7717/peerj.21585/supp-9 Supplemental Information 9 Forest plot showing the agility outcomes for studies with 10- to 26-week plyometric training interventions

10.7717/peerj.21585/supp-10 Supplemental Information 10 Overall forest plot summarizing the pooled effect of plyometric training on agility outcomes across all included agility studies

10.7717/peerj.21585/supp-11 Supplemental Information 11 Forest plot presenting individual study effects and the pooled random-effects estimate for the overall agility analysis

10.7717/peerj.21585/supp-12 Supplemental Information 12 First forest plot page showing jump outcomes for studies with 6- to 7-week plyometric training interventions

10.7717/peerj.21585/supp-13 Supplemental Information 13 Second forest plot page showing additional jump outcomes for the 6- to 7-week intervention subgroup

10.7717/peerj.21585/supp-14 Supplemental Information 14 First forest plot page showing jump outcomes for studies with 8-week plyometric training interventions

10.7717/peerj.21585/supp-15 Supplemental Information 15 Second forest plot page showing additional jump outcomes for the 8-week intervention subgroup

10.7717/peerj.21585/supp-16 Supplemental Information 16 Third forest plot page showing further jump outcomes for the 8-week intervention subgroup

10.7717/peerj.21585/supp-17 Supplemental Information 17 Forest plot showing jump outcomes for studies with 10- to 26-week plyometric training interventions

10.7717/peerj.21585/supp-18 Supplemental Information 18 First forest plot page presenting individual study effects for the overall jump outcome analysis

10.7717/peerj.21585/supp-19 Supplemental Information 19 Second forest plot page presenting additional individual study effects for the overall jump outcome analysis

10.7717/peerj.21585/supp-20 Supplemental Information 20 Third forest plot page presenting further individual study effects for the overall jump outcome analysis

10.7717/peerj.21585/supp-21 Supplemental Information 21 Fourth forest plot page presenting the remaining study effects and pooled estimate for the overall jump outcome analysis

10.7717/peerj.21585/supp-22 Supplemental Information 22 First forest plot page showing sprint outcomes for studies with 6- to 7-week plyometric training interventions

10.7717/peerj.21585/supp-23 Supplemental Information 23 Second forest plot page showing additional sprint outcomes for the 6- to 7-week intervention subgroup

10.7717/peerj.21585/supp-24 Supplemental Information 24 First forest plot page showing sprint outcomes for studies with 8-week plyometric training interventions

10.7717/peerj.21585/supp-25 Supplemental Information 25 Second forest plot page showing additional sprint outcomes for the 8-week intervention subgroup

10.7717/peerj.21585/supp-26 Supplemental Information 26 Forest plot showing sprint outcomes for studies with 10- to 26-week plyometric training interventions

10.7717/peerj.21585/supp-27 Supplemental Information 27 First forest plot page presenting individual study effects for the overall sprint outcome analysis

10.7717/peerj.21585/supp-28 Supplemental Information 28 Second forest plot page presenting additional individual study effects for the overall sprint outcome analysis

10.7717/peerj.21585/supp-29 Supplemental Information 29 Third forest plot page presenting further individual study effects for the overall sprint outcome analysis

10.7717/peerj.21585/supp-30 Supplemental Information 30 Fourth forest plot page presenting the remaining study effects and pooled estimate for the overall sprint outcome analysis

10.7717/peerj.21585/supp-31 Supplemental Information 31 PRISMA checklist

10.7717/peerj.21585/supp-32 Supplemental Information 32 Risk of Bias assessment

## Additional Information and Declarations

### Data Availability

The following information was supplied regarding data availability: This is a Systematic review/Meta-analysis.

## References

[ref-1] Almeida-Neto PFd, De Matos DG, Pinto VCM, Dantas PMS, Cesario TdM, Da Silva LF, Bulhões Correia A, Aidar FJ, De Araújo Tinôco Cabral BG (2020). Can the neuromuscular performance of young athletes be influenced by hormone levels and different stages of puberty?.

[ref-2] Aloui G, Hermassi S, Bartels T, Hayes LD, Bouhafs EG, Chelly MS, Schwesig R (2022). Combined plyometric and short sprint training in U-15 male soccer players: effects on measures of jump, speed, change of direction, repeated sprint, and balance. Frontiers in Physiology.

[ref-3] Aloui G, Hermassi S, Khemiri A, Bartels T, Hayes LD, Bouhafs EG, Chelly MS, Schwesig R (2021). An 8-week program of plyometrics and sprints with changes of direction improved anaerobic fitness in young male soccer players. International Journal of Environmental Research and Public Health.

[ref-4] Anniza M, Iskandar J (2022). The relationship of coordination and agility to the risk of injury in futsal players at Muhammadiyah Cilegon Junior High School. Journal of Applied Health Research and Development.

[ref-5] Asadi A, Arazi H, Young WB, De Villarreal ES (2016). The effects of plyometric training on change-of-direction ability: a meta-analysis. International Journal of Sports Physiology and Performance.

[ref-6] Attene G, Iuliano E, Di Cagno A, Calcagno G, Moalla W, Aquino G, Padulo J (2015). Improving neuromuscular performance in young basketball players: plyometric *vs.* technique training. The Journal of Sports Medicine and Physical Fitness.

[ref-7] Bariya N, Patel K, Pathak I (2019). Test-retest reliability of the 50-meter dash test as a measure of sprinting performance in collegiate sprinters. Journal of Emerging Technologies and Innovative Research.

[ref-8] Bchini S, Hammami N, Selmi T, Zalleg D, Bouassida A (2023). Influence of muscle volume on jumping performance in healthy male and female youth and young adults. BMC Sports Science, Medicine and Rehabilitation.

[ref-9] Beato M, Bianchi M, Coratella G, Merlini M, Drust B (2018). Effects of plyometric and directional training on speed and jump performance in elite youth soccer players. Journal of Strength and Conditioning Research.

[ref-10] Benito-Martínez E, Martínez-Amat A, Lara-Sánchez AJ, Berdejo-Del-Fresno D, Martínez-López EJ (2013). Effect of combined electrostimulation and plyometric training on 30 m dash and triple jump. Journal of Sports Medicine and Physical Fitness.

[ref-11] Berkey CS, Hoaglin DC, Mosteller F, Colditz GA (1995). A random-effects regression model for meta-analysis. Statistics in Medicine.

[ref-12] Bianchi M, Coratella G, Dello Iacono A, Beato M (2019). Comparative effects of single *vs.* double weekly plyometric training sessions on jump, sprint and change of directions abilities of elite youth football players. The Journal of Sports Medicine and Physical Fitness.

[ref-13] Bouguezzi R, Chaabene H, Negra Y, Ramirez-Campillo R, Jlalia Z, Mkaouer B, Hachana Y (2020). Effects of different plyometric training frequencies on measures of athletic performance in prepuberal male soccer players. Journal of Strength and Conditioning Research.

[ref-14] Bouteraa I, Negra Y, Shephard RJ, Chelly MS (2020). Effects of combined balance and plyometric training on athletic performance in female basketball players. Journal of Strength and Conditioning Research.

[ref-15] Buckthorpe M, Della Villa F (2021). Recommendations for plyometric training after ACL reconstruction—a clinical commentary. International Journal of Sports Physical Therapy.

[ref-16] Buğa S, Gencer YG (2022). The effect of plyometric training performed on different surfaces on some performance parameters. Progress in Nutrition.

[ref-17] Chaabene H, Negra Y (2017). The effect of plyometric training volume on athletic performance in prepubertal male soccer players. International Journal of Sports Physiology and Performance.

[ref-18] Chaabene H, Negra Y, Moran J, Prieske O, Sammoud S, Ramirez-Campillo R, Granacher U (2019). Plyometric training improves not only measures of linear speed, power, and change-of-direction speed but also repeated sprint ability in young female handball players. Journal of Strength and Conditioning Research.

[ref-19] Chaouachi M, Granacher U, Makhlouf I, Hammami R, Behm DG, Chaouachi A (2017). Within session sequence of balance and plyometric exercises does not affect training adaptations with youth soccer athletes. Journal of Sports Science and Medicine.

[ref-20] Chelly MS, Hermassi S, Aouadi R, Shephard RJ (2014). Effects of 8-week in-season plyometric training on upper and lower limb performance of elite adolescent handball players. Journal of Strength and Conditioning Research/National Strength & Conditioning Association.

[ref-21] Chtara M, Rouissi M, Haddad M, Chtara H, Chaalali A, Owen A, Chamari K (2017). Specific physical trainability in elite young soccer players: efficiency over 6 weeks’ in-season training. Biology of Sport.

[ref-22] Dardouri W, Khanfir M, Mrayeh M, Alardan S, Zouch M (2023). Normative data of agility T-test as a measure of change of direction speed in children aged 10–11. International Journal of Advanced and Applied Sciences.

[ref-23] Davies MJ, Drury B, Ramirez-Campillo R, Chaabane H, Moran J (2021). Effect of plyometric training and biological maturation on jump and change of direction ability in female youth. The Journal of Strength & Conditioning Research.

[ref-24] De Hoyo M, Gonzalo-Skok O, Sañudo B, Carrascal C, Plaza-Armas JR, Camacho-Candil F, Otero-Esquina C (2016). Comparative effects of in-season full-back squat, resisted sprint training, and plyometric training on explosive performance in U-19 elite soccer players. Journal of Strength and Conditioning Research/National Strength & Conditioning Association.

[ref-25] Faamoe I (2024). Concurrent validity and reliability of the vertical jump and standing broad jump tests in youth.

[ref-26] Falch HN, Haugen ME, Kristiansen EL, Van den Tillaar R (2022). Effect of strength *vs.* plyometric training upon change of direction performance in young female handball players. International Journal of Environmental Research and Public Health.

[ref-27] Fathi A, Hammami R, Moran J, Borji R, Sahli S, Rebai H (2019). Effect of a 16-week combined strength and plyometric training program followed by a detraining period on athletic performance in pubertal volleyball players. Journal of Strength and Conditioning Research.

[ref-28] Ferley DD, Scholten S, Vukovich MD (2020). Combined sprint interval, plyometric, and strength training in adolescent soccer players: effects on measures of speed, strength, power, change of direction, and anaerobic capacity. Journal of Strength and Conditioning Research.

[ref-29] Fernandez-Fernandez J, Saez De Villarreal E, Sanz-Rivas D, Moya M (2016). The effects of 8-week plyometric training on physical performance in young tennis players. Pediatric Exercise Science.

[ref-30] Franco-Márquez F, Rodríguez-Rosell D, González-Suárez JM, Pareja-Blanco F, Mora-Custodio R, Yañez García JM, González-Badillo JJ (2015). Effects of combined resistance training and plyometrics on physical performance in young soccer players. International Journal of Sports Medicine.

[ref-31] Gaamouri N, Hammami M, Cherni Y, Rosemann T, Knechtle B, Chelly MS, Van den Tillaar R (2023). The effects of 10-week plyometric training program on athletic performance in youth female handball players. Frontiers in Sports and Active Living.

[ref-32] Gonzalo-Skok O, Sánchez-Sabaté J, Izquierdo-Lupón L, Sáez De Villarreal E (2019). Influence of force-vector and force application plyometric training in young elite basketball players. European Journal of Sport Science.

[ref-33] Guadalupe-Grau A, Perez-Gomez J, Olmedillas H, Chavarren J, Dorado C, Santana A, Serrano-Sanchez JA, Calbet JA (2009). Strength training combined with plyometric jumps in adults: sex differences in fat-bone axis adaptations. Journal of Applied Physiology (1985).

[ref-34] Guthold R, Stevens GA, Riley LM, Bull FC (2020). Global trends in insufficient physical activity among adolescents: a pooled analysis of 298 population-based surveys with 1⋅6 million participants. The Lancet Child & Adolescent Health.

[ref-35] Hammami M, Bragazzi NL, Hermassi S, Gaamouri N, Aouadi R, Shephard RJ, Chelly MS (2020a). The effect of a sand surface on physical performance responses of junior male handball players to plyometric training. BMC Sports Science, Medicine and Rehabilitation.

[ref-36] Hammami M, Gaamouri N, Ramirez-Campillo R, Shephard RJ, Bragazzi NL, Chelly MS, Knechtle B, Gaied S (2021). Effects of high-intensity interval training and plyometric exercise on the physical fitness of junior male handball players. European Review for Medical and Pharmacological Sciences.

[ref-37] Hammami M, Gaamouri N, Shephard RJ, Chelly MS (2019). Effects of contrast strength *vs.* plyometric training on lower-limb explosive performance, ability to change direction and neuromuscular adaptation in soccer players. Journal of Strength and Conditioning Research.

[ref-38] Hammami M, Gaamouri N, Suzuki K, Aouadi R, Shephard RJ, Chelly MS (2020b). Effects of unloaded *vs.* ankle-loaded plyometric training on the physical fitness of U-17 male soccer players. International Journal of Environmental Research and Public Health.

[ref-39] Hammami M, Gaamouri N, Suzuki K, Shephard RJ, Chelly MS (2020c). Effects of upper and lower limb plyometric training program on components of physical performance in young female handball players. Frontiers in Physiology.

[ref-40] Harries SK, Lubans DR, Buxton A, MacDougall TH, Callister R (2018). Effects of 12-week resistance training on sprint and jump performances in competitive adolescent rugby union players. The Journal of Strength & Conditioning Research.

[ref-41] Hayashino Y, Noguchi Y, Fukui T (2005). Systematic evaluation and comparison of statistical tests for publication bias. Journal of Epidemiology.

[ref-42] Hernandez-Martinez J, Guzman-Muñoz E, Ramirez-Campillo R, Herrera-Valenzuela T, Magnani Branco BH, Avila-Valencia S, Luis Carter-Beltran J, Aravena-Sagardia P, Méndez-Cornejo J, Valdés-Badilla P (2023). Effects of different plyometric training frequencies on physical performance in youth male volleyball players: a randomized trial. Frontiers in Physiology.

[ref-43] Higgins JPT, Thompson SG (2002). Quantifying heterogeneity in a meta-analysis. Statistics in Medicine.

[ref-44] Idrizovic K, Gjinovci B, Sekulic D, Uljevic O, João PV, Spasic M, Sattler T (2017). The effects of 3-month skill-based and plyometric conditioning on fitness parameters in junior female volleyball players. Pediatric Exercise Science.

[ref-45] Jlid MC, Racil G, Coquart J, Paillard T, Bisciotti GN, Chamari K (2019). Multidirectional plyometric training: very efficient way to improve vertical jump performance, change of direction performance and dynamic postural control in young soccer players. Frontiers in Physiology.

[ref-46] Kobel R, Pereira LA, Zanetti V, Ramirez-Campillo R, Loturco I (2017). Effects of unloaded *vs.* loaded plyometrics on speed and power performance of elite young soccer players. Frontiers in Physiology.

[ref-47] Kryeziu AR, Iseni A, Teodor DF, Croitoru H, Badau D (2023). Effect of 12 weeks of the plyometric training program model on speed and explosive strength abilities in adolescents. Applied Sciences-Basel.

[ref-48] Kurt C, Canli U, Erdaş SE, Poli L, Carvutto R, Cataldi S, Fischetti F, Greco G (2023). Effectiveness of vertical *versus* horizontal plyometric training on stretch-shortening cycle performance enhancement in adolescent soccer players. Healthcare.

[ref-49] Liberati A, Altman DG, Tetzlaff J, Mulrow C, Gøtzsche PC, Ioannidis JP, Clarke M, Devereaux PJ, Kleijnen J, Moher D (2009). The PRISMA statement for reporting systematic reviews and meta-analyses of studies that evaluate healthcare interventions: explanation and elaboration. BMJ.

[ref-50] Liu GY, Wang XS, Xu Q (2024). Microdosing plyometric training enhances jumping performance, reactive strength index, and acceleration among youth soccer players: a randomized controlled study design. Journal of Sports Science and Medicine.

[ref-51] Lloyd RS, Radnor JM, De Ste Croix MBA, Cronin JB, Oliver JL (2016). Changes in sprint and jump performances after traditional, plyometric, and combined resistance training in male youth pre- and post-peak height velocity. Journal of Strength and Conditioning Research.

[ref-52] Mackala K, Vodičar J, Žvan M, Križaj J, Stodolka J, Rauter S, Šimenko J, Čoh Ijoer M (2020). Evaluation of the pre-planned and non-planed agility performance: comparison between individual and team sports. International Journal of Environmental Research and Public Health.

[ref-53] Makhlouf I, Chaouachi A, Chaouachi M, Othman AB, Granacher U, Behm DG (2018). Combination of agility and plyometric training provides similar training benefits as combined balance and plyometric training in young soccer players. Frontiers in Physiology.

[ref-54] Markovic G (2007). Does plyometric training improve vertical jump height? A meta-analytical review. British Journal of Sports Medicine.

[ref-55] Marzouki H, Sbai S, Ouergui I, Selmi O, Andrade MS, Bouhlel E, Thuany M, Weiss K, Nikolaidis PT, Knechtle B (2023). Effects of biological age on athletic adaptations to combined plyometric and sprint with change of direction with ball training in youth soccer players. Biology.

[ref-56] McKinlay BJ, Wallace P, Dotan R, Long D, Tokuno C, Gabriel DA, Falk B (2018). Effects of plyometric and resistance training on muscle strength, explosiveness, and neuromuscular function in young adolescent soccer players. Journal of Strength and Conditioning Research.

[ref-57] Meszler B, Váczi M (2019). Effects of short-term in-season plyometric training in adolescent female basketball players. Physiology International.

[ref-58] Meylan C, Malatesta D (2009). Effects of in-season plyometric training within soccer practice on explosive actions of young players. Journal of Strength and Conditioning Research/National Strength & Conditioning Association.

[ref-59] Moran J, Sandercock G, Ramirez-Campillo R, Clark CCT, Fernandes JFT, Drury B (2018). A meta-analysis of resistance training in female youth: its effect on muscular strength, and shortcomings in the literature. Sports Medicine.

[ref-60] Moran J, Sandercock GRH, Ramírez-Campillo R, Todd O, Collison J, Parry DA (2016). Maturation-related effect of low-dose plyometric training on performance in youth hockey players. Pediatric Exercise Science.

[ref-61] Mănescu DC (2025). Computational analysis of neuromuscular adaptations to strength and plyometric training: an integrated modeling study. Sports.

[ref-62] Negra Y, Chaabene H, Fernandez-Fernandez J, Sammoud S, Bouguezzi R, Prieske O, Granacher U (2020). Short-term plyometric jump training improves repeated-sprint ability in prepuberal male soccer players. Journal of Strength and Conditioning Research.

[ref-63] Negra Y, Chaabene H, Sammoud S, Bouguezzi R, Abbes MA, Hachana Y, Granacher U (2017a). Effects of plyometric training on physical fitness in prepuberal soccer athletes. International Journal of Sports Medicine.

[ref-64] Negra Y, Chaabene H, Sammoud S, Bouguezzi R, Mkaouer B, Hachana Y, Granacher U (2017b). Effects of plyometric training on components of physical fitness in prepuberal male soccer athletes: the role of surface instability. Journal of Strength and Conditioning Research.

[ref-65] Norgeot F, Fouré A (2024). Effects of vertical and horizontal plyometric training on jump performances and sprint force-velocity profile in young elite soccer players. European Journal of Applied Physiology.

[ref-66] Novak D, Loncar I, Sinkovic F, Barbaros P, Milanovic L (2023). Effects of plyometric training with resistance bands on neuromuscular characteristics in junior tennis players. International Journal of Environmental Research and Public Health.

[ref-67] Nygaard Falch H, Guldteig Rædergård H, Van den Tillaar R (2019). Effect of different physical training forms on change of direction ability: a systematic review and meta-analysis. Sports Medicine—Open.

[ref-68] Oliver JL, Ramachandran AK, Singh U, Ramirez-Campillo R, Lloyd RS (2024). The effects of strength, plyometric and combined training on strength, power and speed characteristics in high-level, highly trained male youth soccer players: a systematic review and meta-analysis. Sports Medicine.

[ref-69] Padrón-Cabo A, Lorenzo-Martínez M, Pérez-Ferreirós A, Costa PB, Rey E (2021). Effects of plyometric training with agility ladder on physical fitness in youth soccer players. International Journal of Sports Medicine.

[ref-70] Page MJ, McKenzie JE, Bossuyt PM, Boutron I, Hoffmann TC, Mulrow CD, Shamseer L, Tetzlaff JM, Akl EA, Brennan SE, Chou R, Glanville J, Grimshaw JM, Hróbjartsson A, Lalu MM, Li T, Loder EW, Mayo-Wilson E, McDonald S, McGuinness LA, Stewart LA, Thomas J, Tricco AC, Welch VA, Whiting P, Moher D (2021). The PRISMA 2020 statement: an updated guideline for reporting systematic reviews. BMJ.

[ref-71] Palma-Muñoz I, Ramírez-Campillo R, Azocar-Gallardo J, Álvarez C, Asadi A, Moran J, Chaabene H (2021). Effects of progressed and nonprogressed volume-based overload plyometric training on components of physical fitness and body composition variables in youth male basketball players. Journal of Strength and Conditioning Research.

[ref-72] Paul DJ, Gabbett TJ, Nassis GP (2016). Agility in team sports: testing, training and factors affecting performance. Sports Medicine.

[ref-73] Potdevin FJ, Alberty ME, Chevutschi A, Pelayo P, Sidney MC (2011). Effects of a 6-week plyometric training program on performances in pubescent swimmers. Journal of Strength and Conditioning Research.

[ref-74] Ramirez-Campillo R, Álvarez C, García-Pinillos F, García-Ramos A, Loturco I, Chaabene H, Granacher U (2020a). Effects of combined surfaces *vs.* single-surface plyometric training on soccer players’ physical fitness. Journal of Strength and Conditioning Research.

[ref-75] Ramirez-Campillo R, Alvarez C, García-Pinillos F, Gentil P, Moran J, Pereira LA, Loturco I (2019). Effects of plyometric training on physical performance of young male soccer players: potential effects of different drop jump heights. Pediatric Exercise Science.

[ref-76] Ramirez-Campillo R, Alvarez C, García-Pinillos F, Sanchez-Sanchez J, Yanci J, Castillo D, Loturco I, Chaabene H, Moran J, Izquierdo M (2018). Optimal reactive strength index: is it an accurate variable to optimize plyometric training effects on measures of physical fitness in young soccer players?. Journal of Strength and Conditioning Research.

[ref-77] Ramirez-Campillo R, Alvarez C, Gentil P, Loturco I, Sanchez-Sanchez J, Izquierdo M, Moran J, Nakamura FY, Chaabene H, Granacher U (2020b). Sequencing effects of plyometric training applied before or after regular soccer training on measures of physical fitness in young players. Journal of Strength and Conditioning Research.

[ref-78] Ramirez-Campillo R, Andrade DC, Nikolaidis PT, Moran J, Clemente FM, Chaabene H, Comfort P (2020c). Effects of plyometric jump training on vertical jump height of volleyball players: a systematic review with meta-analysis of randomized-controlled trial. Journal of Sports Science and Medicine.

[ref-79] Ramirez-Campillo R, Álvarez C, García-Pinillos F, García-Ramos A, Loturco I, Chaabene H, Granacher U (2022). The effects of plyometric jump training on physical fitness attributes in basketball players: a meta-analysis. Journal of Sport and Health Science.

[ref-80] Ramírez-dela Cruz M, Bravo-Sánchez A, Esteban-García P, Jiménez F, Abián-Vicén J (2022). Effects of plyometric training on lower body muscle architecture, tendon structure, stiffness and physical performance: a systematic review and meta-analysis. Sports Medicine—Open.

[ref-81] Roberts BM, Nuckols G, Krieger JW (2020). Sex differences in resistance training: a systematic review and meta-analysis. The Journal of Strength & Conditioning Research.

[ref-82] Rubley MD, Haase AC, Holcomb WR, Girouard TJ, Tandy RD (2011). The effect of plyometric training on power and kicking distance in female adolescent soccer players. Journal of Strength and Conditioning Research.

[ref-83] Sáez De Villarreal E, Molina JG, De Castro-Maqueda G, Gutiérrez-Manzanedo JV (2021). Effects of plyometric, strength and change of direction training on high-school basketball player’s physical fitness. Journal of Human Kinetics.

[ref-84] Sáez de Villarreal E, Suarez-Arrones L, Requena B, Haff GG, Ferrete C (2015). Effects of plyometric and sprint training on physical and technical skill performance in adolescent soccer players. Journal of Strength and Conditioning Research/National Strength & Conditioning Association.

[ref-85] Sammoud S, Negra Y, Bouguezzi R, Ramirez-Campillo R, Moran J, Bishop C, Chaabene H (2024). Effects of plyometric jump training on measures of physical fitness and lower-limb asymmetries in prepubertal male soccer players: a randomized controlled trial. BMC Sports Science, Medicine and Rehabilitation.

[ref-86] Sánchez M, Sanchez-Sanchez J, Nakamura FY, Clemente FM, Romero-Moraleda B, Ramirez-Campillo R (2020). Effects of plyometric jump training in female soccer player’s physical fitness: a systematic review with meta-analysis. International Journal of Environmental Research and Public Health.

[ref-87] Santos EJAM, Janeira MAAS (2011). The effects of plyometric training followed by detraining and reduced training periods on explosive strength in adolescent male basketball players. Journal of Strength and Conditioning Research.

[ref-88] Slimani M, Chamari K, Miarka B, Del Vecchio FB, Cheour F (2016). Effects of plyometric training on physical fitness in team sport athletes: a systematic review. Journal of Human Kinetics.

[ref-89] Söderström T, Fahlén J, Ferry M, Yu J (2018). Athletic ability in childhood and adolescence as a predictor of participation in non-elite sports in young adulthood. Sports in Scoiety.

[ref-90] Söhnlein Q, Müller E, Stöggl TL (2014). The effect of 16-week plyometric training on explosive actions in early to mid-puberty elite soccer players. Journal of Strength and Conditioning Research/National Strength & Conditioning Association.

[ref-91] Söyler M, Zileli R, Çingöz YE, Kılınçarslan G, Kayantaş İ, Altuğ T, Asan S, Şahin M, Gürkan AC (2024). The effect of high-intensity plyometric training on anaerobic performance parameters: a pilot study in U17 elite A league. PeerJ.

[ref-92] Tantry TP, Karanth H, Shetty PK, Kadam DJKJoA (2021). Self-learning software tools for data analysis in meta-analysis. Korean Journal of Anesthesiology.

[ref-93] Thaqi A, Berisha M, Hoxha S (2020). The effect of plyometric training on the power-related factors of children aged 16 years-old. Progress in Nutrition.

[ref-94] Vera-Assaoka T, Ramirez-Campillo R, Alvarez C, Garcia-Pinillos F, Moran J, Gentil P, Behm D (2020). Effects of maturation on physical fitness adaptations to plyometric drop jump training in male youth soccer players. Journal of Strength and Conditioning Research.

[ref-95] Wang X, Zhang K, Samsudin SB, Hassan MZB, Yaakob S, Dong D (2024). Effects of plyometric training on physical fitness attributes in handball players: a systematic review and meta-analysis. Journal of Sports Science and Medicine.

[ref-96] World Health Organization (2018). Adolescent health and development.

[ref-97] Zheng T, Kong R, Liang X, Huang Z, Luo X, Zhang X, Xiao Y (2025). Effects of plyometric training on jump, sprint, and change of direction performance in adolescent soccer player: a systematic review with meta-analysis. PLOS ONE.

