# Supplementary material for: The effects of plyometric training on adolescent sports performance: a systematic review and meta-analysis
Source: PeerJ. 2026 Jul 23;14:e21585. doi: 10.7717/peerj.21585 (PMC13401847; doi:10.7717/peerj.21585)
Supplement: Supplemental Information 1 [file peerj-14-21585-s001.pdf]

**Supplementary File 1.** Search strategy.

| Database | Search Equation | Records retrieved | |
|---|---|---|---|
| | | January 1 ,2025 | January 30, 2026 |
| PubMed | ("Plyometric Exercise"[Mesh] OR plyometric*[tiab] OR "plyometric exercise"[tiab] OR "plyometric exercises"[tiab] OR "plyometric training"[tiab] OR "plyometric trainings"[tiab] OR "plyometric drill"[tiab] OR "plyometric drills"[tiab] OR "stretch-shortening exercise"[tiab] OR "stretch-shortening exercises"[tiab] OR "stretch shortening exercise"[tiab] OR "stretch shortening exercises"[tiab] OR "stretch-shortening drill"[tiab] OR "stretch-shortening drills"[tiab] OR "stretch shortening drill"[tiab] OR "stretch shortening drills"[tiab] OR "stretch-shortening cycle"[tiab] OR "stretch shortening cycle"[tiab] OR "stretch-shortening cycle exercise"[tiab] OR "stretch-shortening cycle exercises"[tiab] OR "stretch shortening cycle exercise"[tiab] OR "stretch shortening cycle exercises"[tiab]) AND ("Adolescent"[Mesh] OR "Child"[Mesh] OR adolescen*[tiab] OR youth*[tiab] OR teen*[tiab] OR teenage*[tiab] OR teenager*[tiab] OR child*[tiab] OR boy*[tiab] OR girl*[tiab] OR junior*[tiab] OR pubertal[tiab] OR prepubertal[tiab]) AND ("Athletic Performance"[Mesh] OR "Physical Functional Performance"[Mesh] OR "Exercise Tolerance"[Mesh] OR "Physical Endurance"[Mesh] OR "Motor Skills"[Mesh] OR "Pliability"[Mesh] OR "athletic performance"[tiab] OR "athletic performances"[tiab] OR "sports performance"[tiab] OR "sports performances"[tiab] OR "physical functional performance"[tiab] OR "functional performance"[tiab] OR "physical performance"[tiab] OR "motor skill"[tiab] OR "motor skills"[tiab] OR "exercise tolerance"[tiab] OR "physical endurance"[tiab] OR "physical stamina"[tiab] OR flexibility[tiab] OR pliability[tiab] OR performance[tiab] OR power[tiab] OR agility[tiab] OR speed[tiab] OR coordination[tiab] OR "strength endurance"[tiab] OR jump*[tiab] OR "vertical jump"[tiab] OR "countermovement jump"[tiab] OR "squat jump"[tiab] OR "standing long jump"[tiab] OR sprint*[tiab] OR acceleration[tiab] OR "change of direction"[tiab] OR "change-of-direction"[tiab] OR COD[tiab]) | 434 | 49 |

| | | | |
|---|---|---|---|
| Embase | ('plyometric exercise'/exp OR plyometric*:ti,ab,kw OR 'plyometric exercise':ti,ab,kw OR 'plyometric exercises':ti,ab,kw OR 'plyometric training':ti,ab,kw OR 'plyometric trainings':ti,ab,kw OR 'plyometric drill':ti,ab,kw OR 'plyometric drills':ti,ab,kw OR 'stretch-shortening exercise':ti,ab,kw OR 'stretch-shortening exercises':ti,ab,kw OR 'stretch shortening exercise':ti,ab,kw OR 'stretch shortening exercises':ti,ab,kw OR 'stretch-shortening drill':ti,ab,kw OR 'stretch-shortening drills':ti,ab,kw OR 'stretch shortening drill':ti,ab,kw OR 'stretch shortening drills':ti,ab,kw OR 'stretch-shortening cycle':ti,ab,kw OR 'stretch shortening cycle':ti,ab,kw OR 'stretch-shortening cycle exercise':ti,ab,kw OR 'stretch-shortening cycle exercises':ti,ab,kw OR 'stretch shortening cycle exercise':ti,ab,kw OR 'stretch shortening cycle exercises':ti,ab,kw) AND ('adolescent'/exp OR 'child'/exp OR adolescen*:ti,ab,kw OR youth*:ti,ab,kw OR teen*:ti,ab,kw OR teenage*:ti,ab,kw OR teenager*:ti,ab,kw OR child*:ti,ab,kw OR boy*:ti,ab,kw OR girl*:ti,ab,kw OR junior*:ti,ab,kw OR pubertal:ti,ab,kw OR prepubertal:ti,ab,kw) AND ('athletic performance'/exp OR 'physical performance'/exp OR 'exercise tolerance'/exp OR 'physical endurance'/exp OR 'motor skill'/exp OR 'athletic performance':ti,ab,kw OR 'athletic performances':ti,ab,kw OR 'sports performance':ti,ab,kw OR 'sports performances':ti,ab,kw OR 'physical functional performance':ti,ab,kw OR 'functional performance':ti,ab,kw OR 'physical performance':ti,ab,kw OR 'motor skill':ti,ab,kw OR 'motor skills':ti,ab,kw OR 'exercise tolerance':ti,ab,kw OR 'physical endurance':ti,ab,kw OR 'physical stamina':ti,ab,kw OR flexibility:ti,ab,kw OR pliability:ti,ab,kw OR performance:ti,ab,kw OR power:ti,ab,kw OR agility:ti,ab,kw OR speed:ti,ab,kw OR coordination:ti,ab,kw OR 'strength endurance':ti,ab,kw OR jump*:ti,ab,kw OR 'vertical jump':ti,ab,kw OR 'countermovement jump':ti,ab,kw OR 'squat jump':ti,ab,kw OR 'standing long jump':ti,ab,kw OR sprint*:ti,ab,kw OR acceleration:ti,ab,kw OR 'change of direction':ti,ab,kw OR 'change-of-direction':ti,ab,kw OR COD:ti,ab,kw) | 408 | 17 |

| | | | |
|---|---|---|---|
| Web of Science | TS=(plyometric* OR "plyometric exercise" OR "plyometric exercises" OR "plyometric training" OR "plyometric trainings" OR "plyometric drill" OR "plyometric drills" OR "stretch-shortening exercise" OR "stretch-shortening exercises" OR "stretch shortening exercise" OR "stretch shortening exercises" OR "stretch-shortening drill" OR "stretch-shortening drills" OR "stretch shortening drill" OR "stretch shortening drills" OR "stretch-shortening cycle" OR "stretch shortening cycle" OR "stretch-shortening cycle exercise" OR "stretch-shortening cycle exercises" OR "stretch shortening cycle exercise" OR "stretch shortening cycle exercises") AND TS=(adolescen* OR youth* OR teen* OR teenage* OR teenager* OR child* OR boy* OR girl* OR junior* OR pubertal OR prepubertal) AND TS=("athletic performance" OR "athletic performances" OR "sports performance" OR "sports performances" OR "physical functional performance" OR "functional performance" OR "physical performance" OR "motor skill" OR "motor skills" OR "exercise tolerance" OR "physical endurance" OR "physical stamina" OR flexibility OR pliability OR performance OR power OR agility OR speed OR coordination OR "strength endurance" OR jump* OR "vertical jump" OR "countermovement jump" OR "squat jump" OR "standing long jump" OR sprint* OR acceleration OR "change of direction" OR "change-of-direction" OR COD) | 209 | 15 |
| | TOTAL | 1051 | 81 |
