# Supplementary figures and images for: The effects of plyometric training on adolescent sports performance: a systematic review and meta-analysis
Source: PeerJ. 2026 Jul 23;14:e21585. doi: 10.7717/peerj.21585 (PMC13401847; doi:10.7717/peerj.21585)

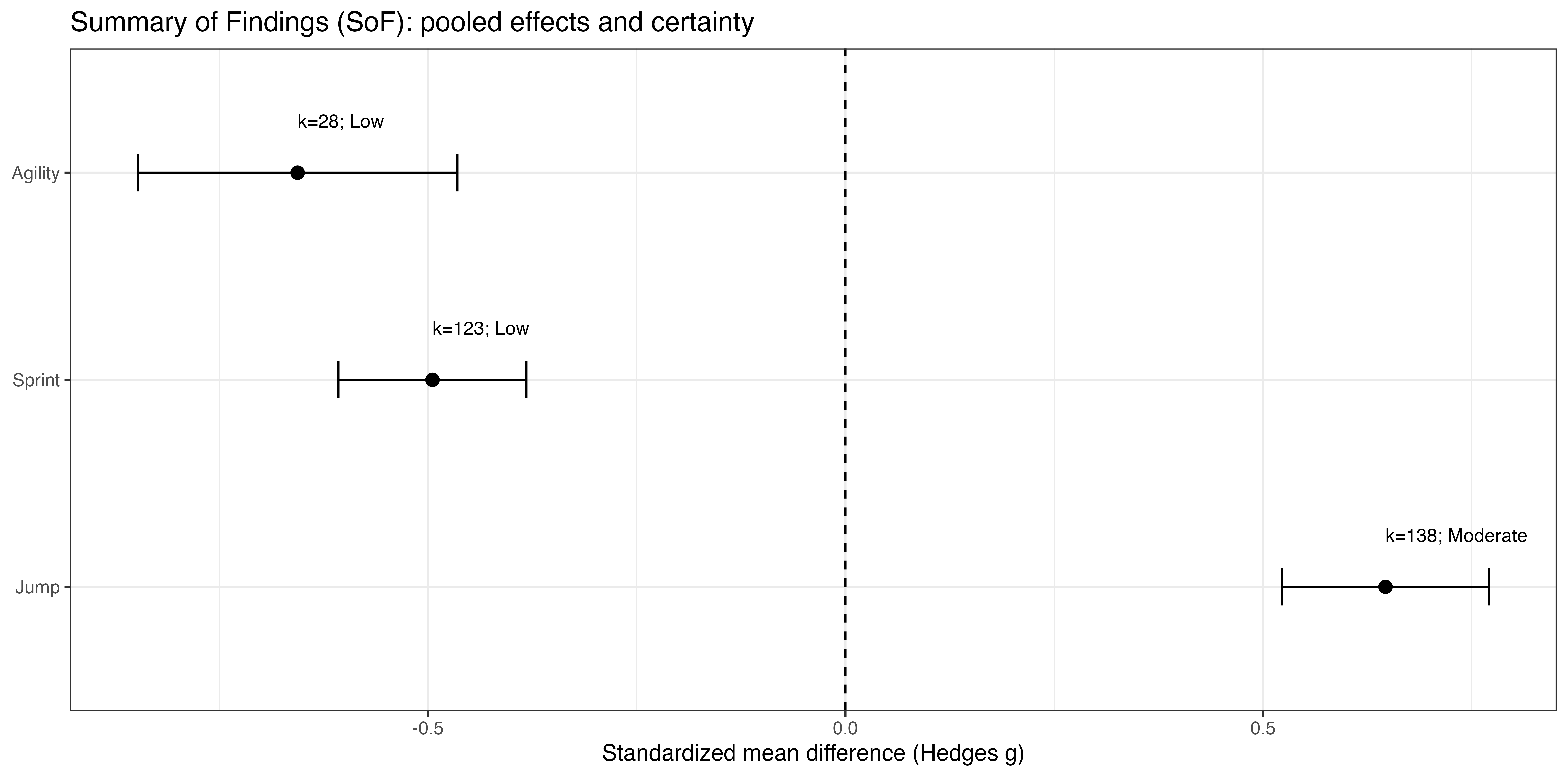

Supplement: Supplemental Information 2 [file peerj-14-21585-s002.png]

Agility (subgroup: dur2)

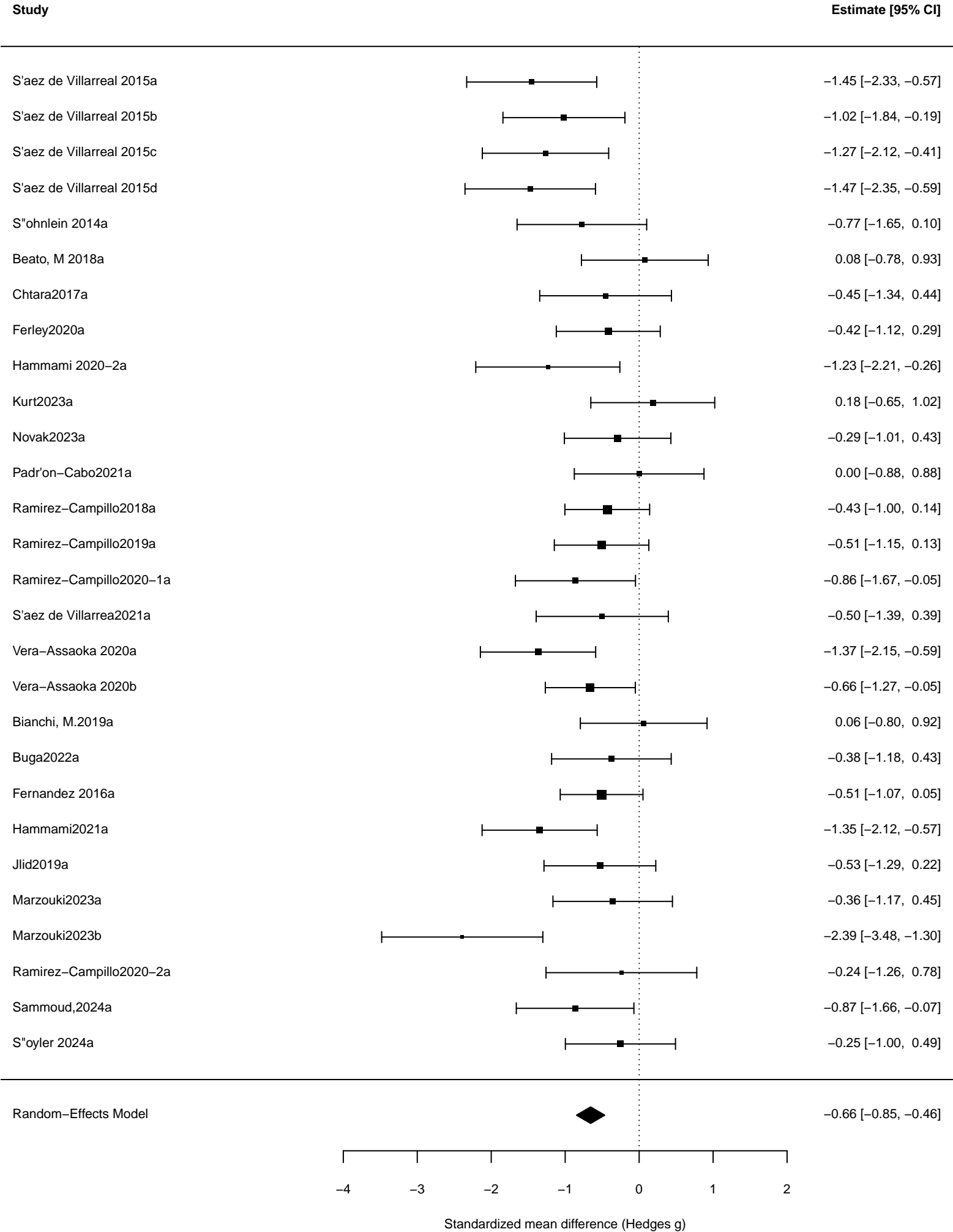

Supplement: Supplemental Information 6 [file peerj-14-21585-s006.pdf]

## Study

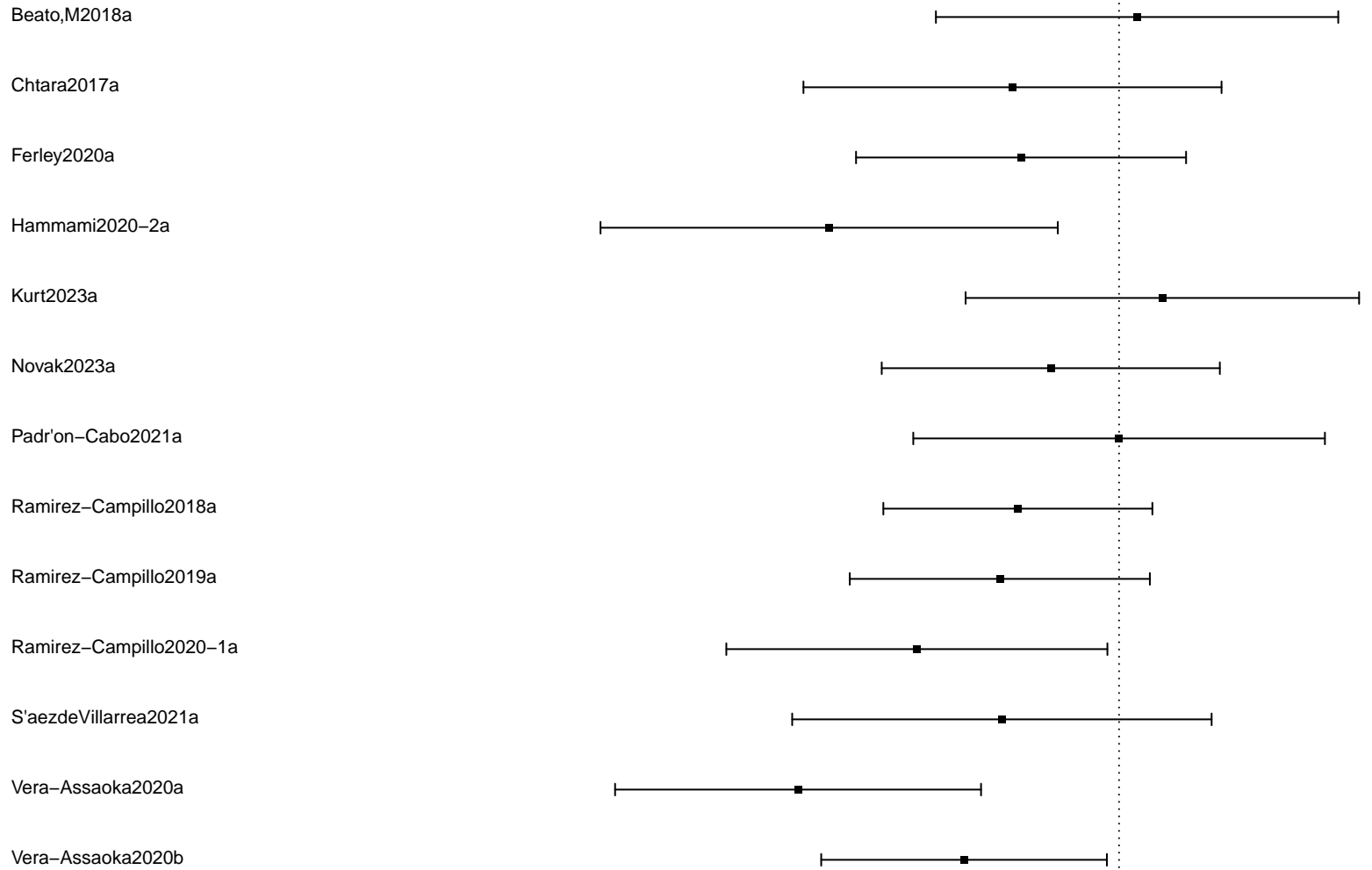

Overall—Effects Model

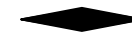

-3.00 -2.00 -1.00 0.00 1.00

Standardized mean difference (Hedges g)

Supplement: Supplemental Information 7 [file peerj-14-21585-s007.pdf]

Study

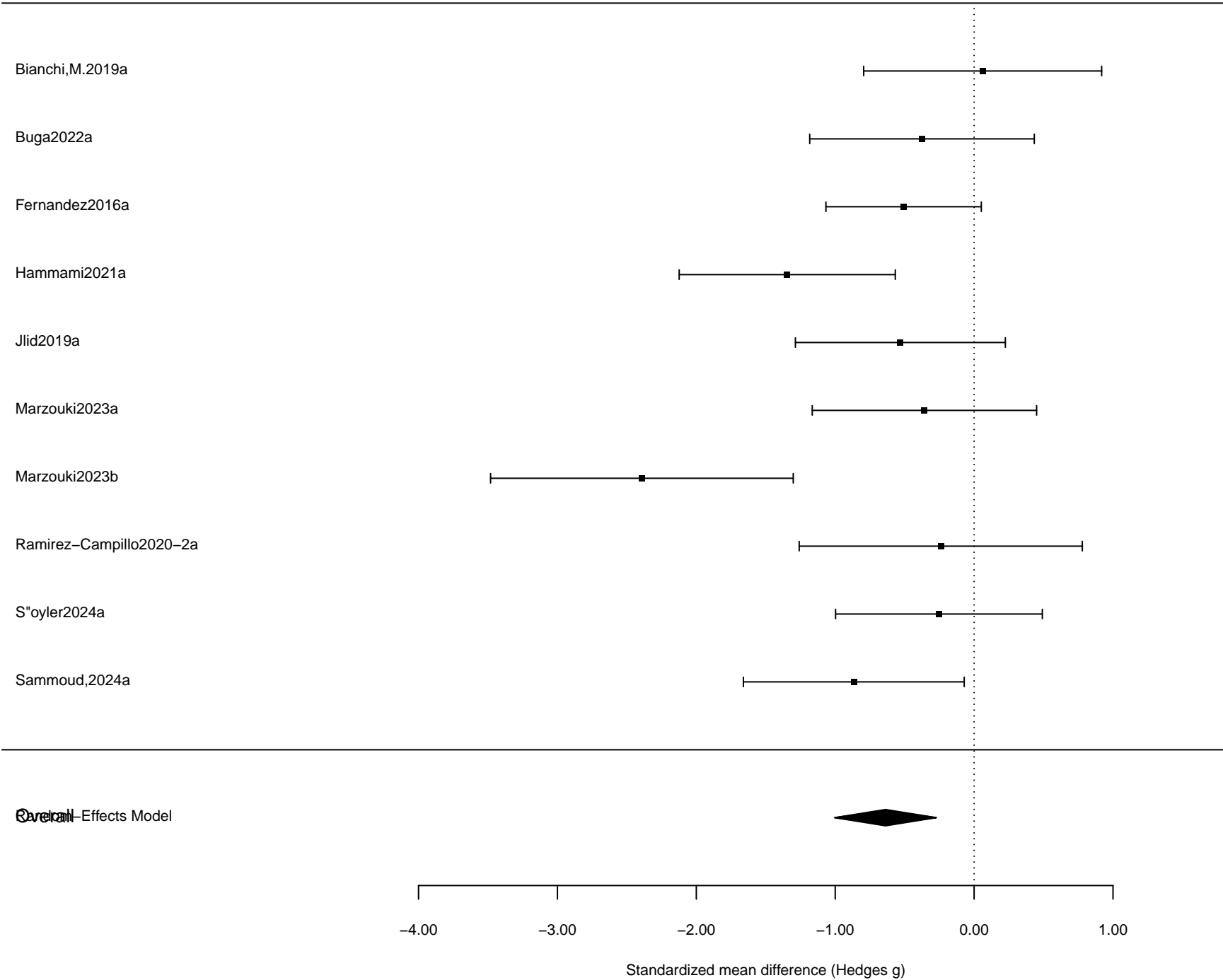

Supplement: Supplemental Information 8 [file peerj-14-21585-s008.pdf]

# Agility

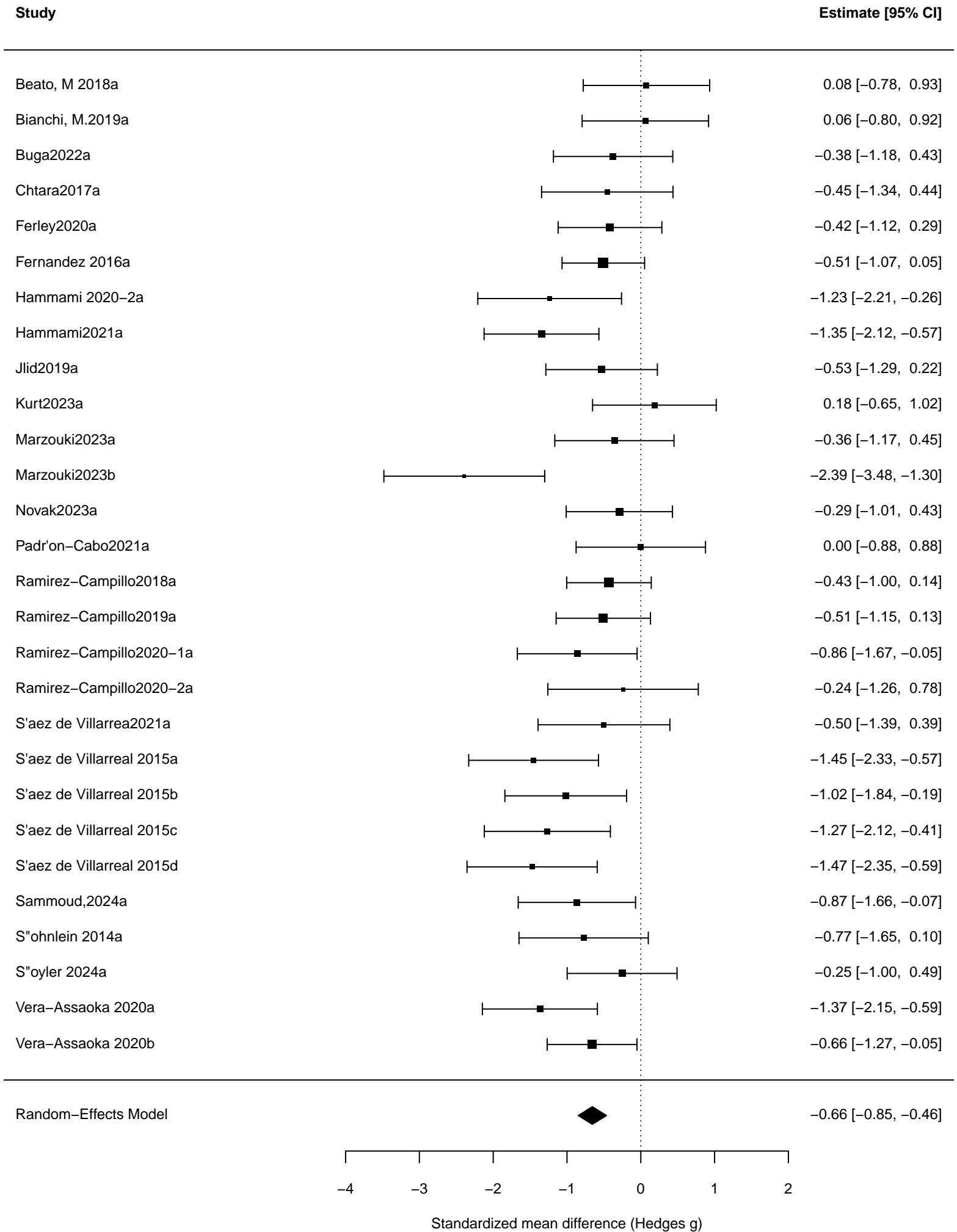

Supplement: Supplemental Information 10 [file peerj-14-21585-s010.pdf]

Study

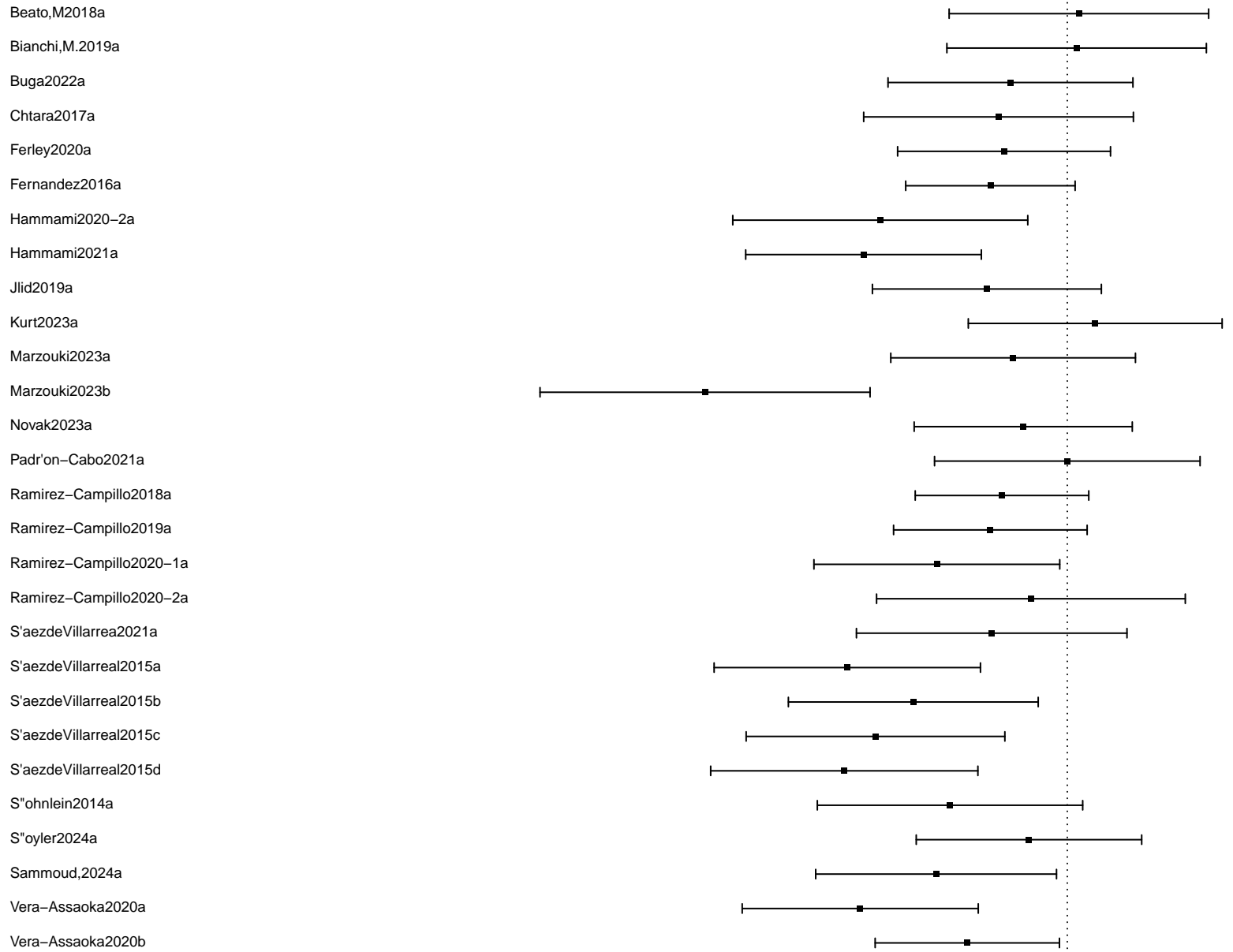

Overall Effects Model

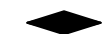

-4.00 -3.00 -2.00 -1.00 0.00 1.00

Standardized mean difference (Hedges g)

Supplement: Supplemental Information 11 [file peerj-14-21585-s011.pdf]

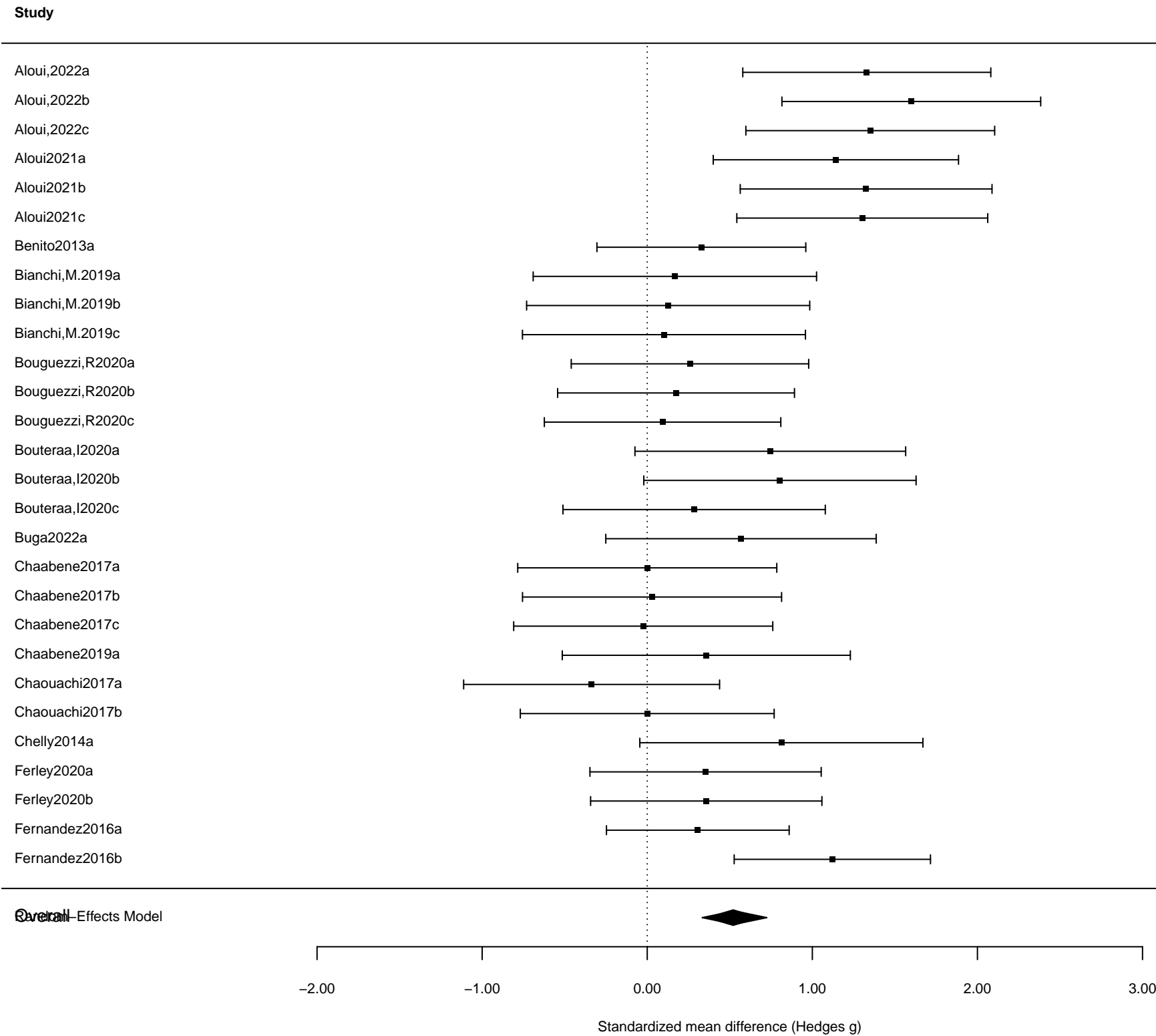

Supplement: Supplemental Information 14 [file peerj-14-21585-s014.pdf]

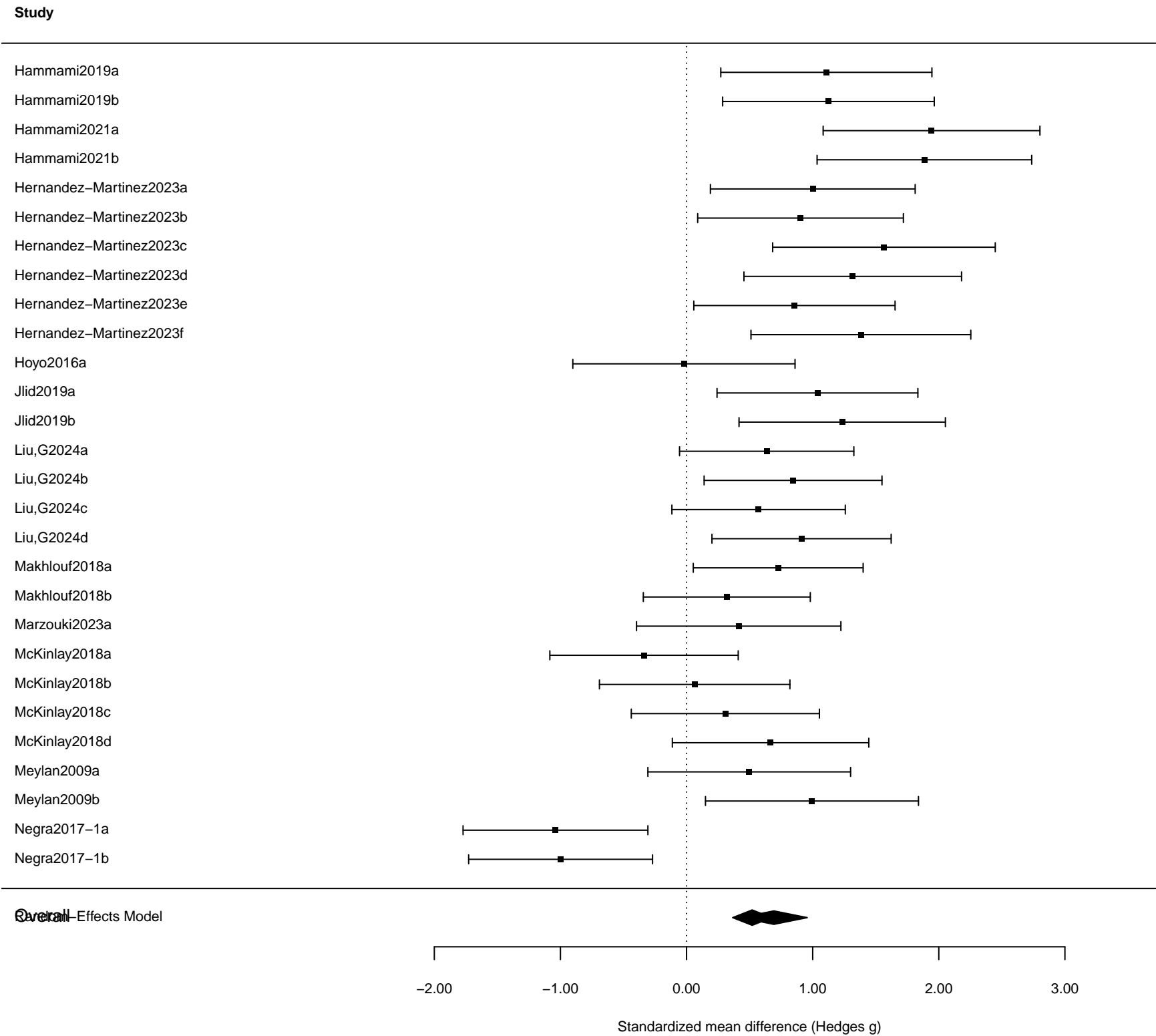

Supplement: Supplemental Information 15 [file peerj-14-21585-s015.pdf]

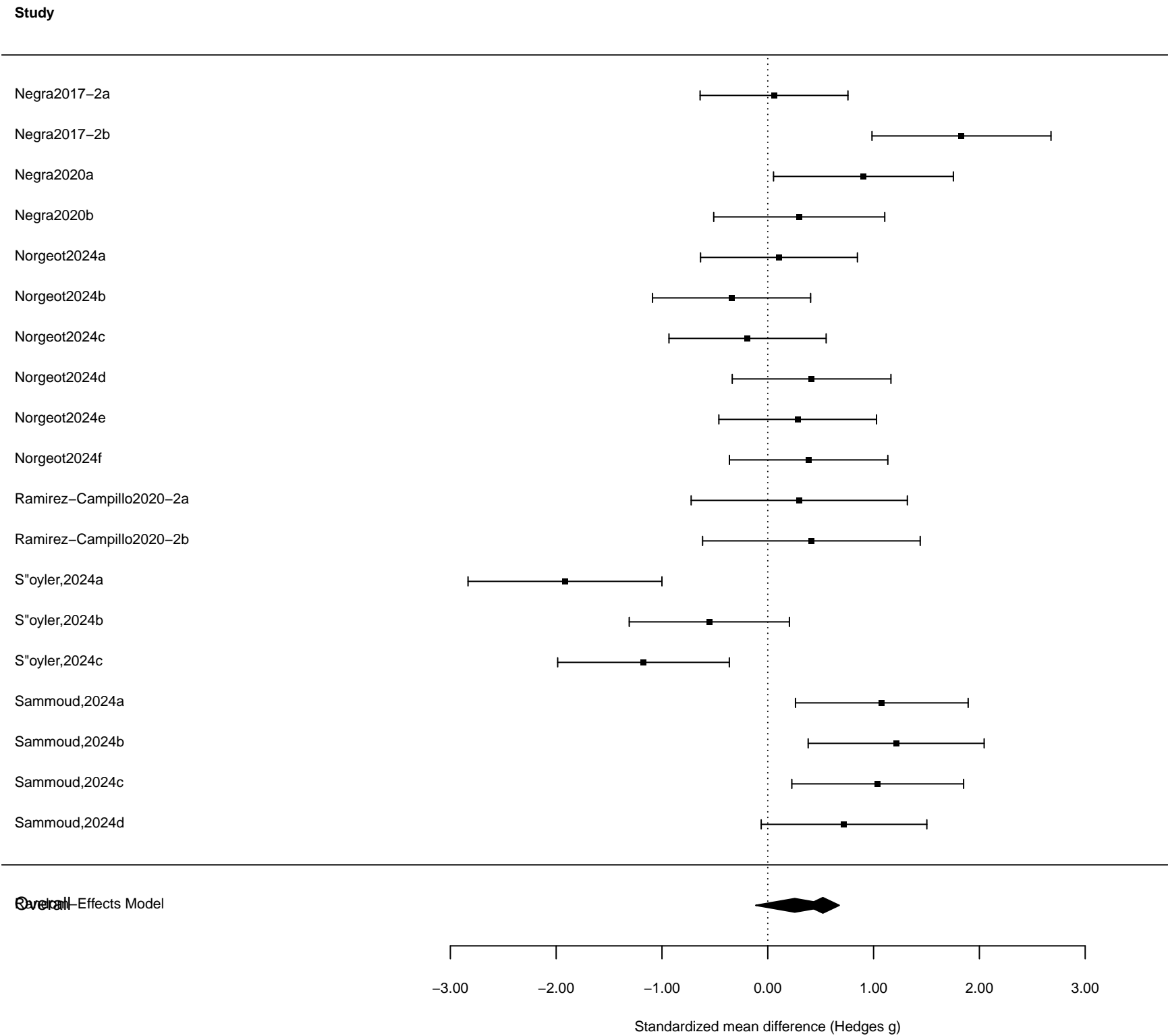

Supplement: Supplemental Information 16 [file peerj-14-21585-s016.pdf]

Study

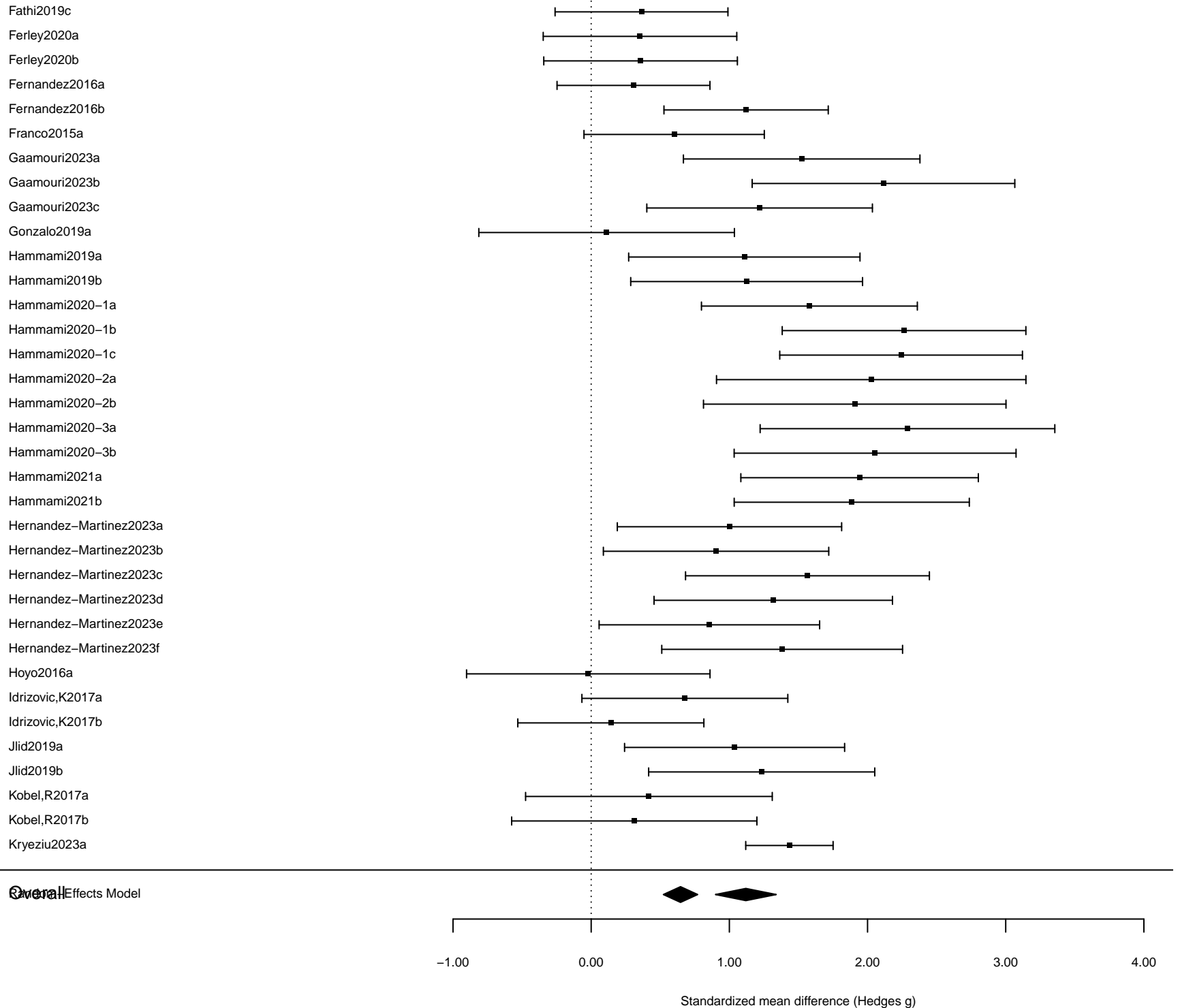

Supplement: Supplemental Information 19 [file peerj-14-21585-s019.pdf]

Study

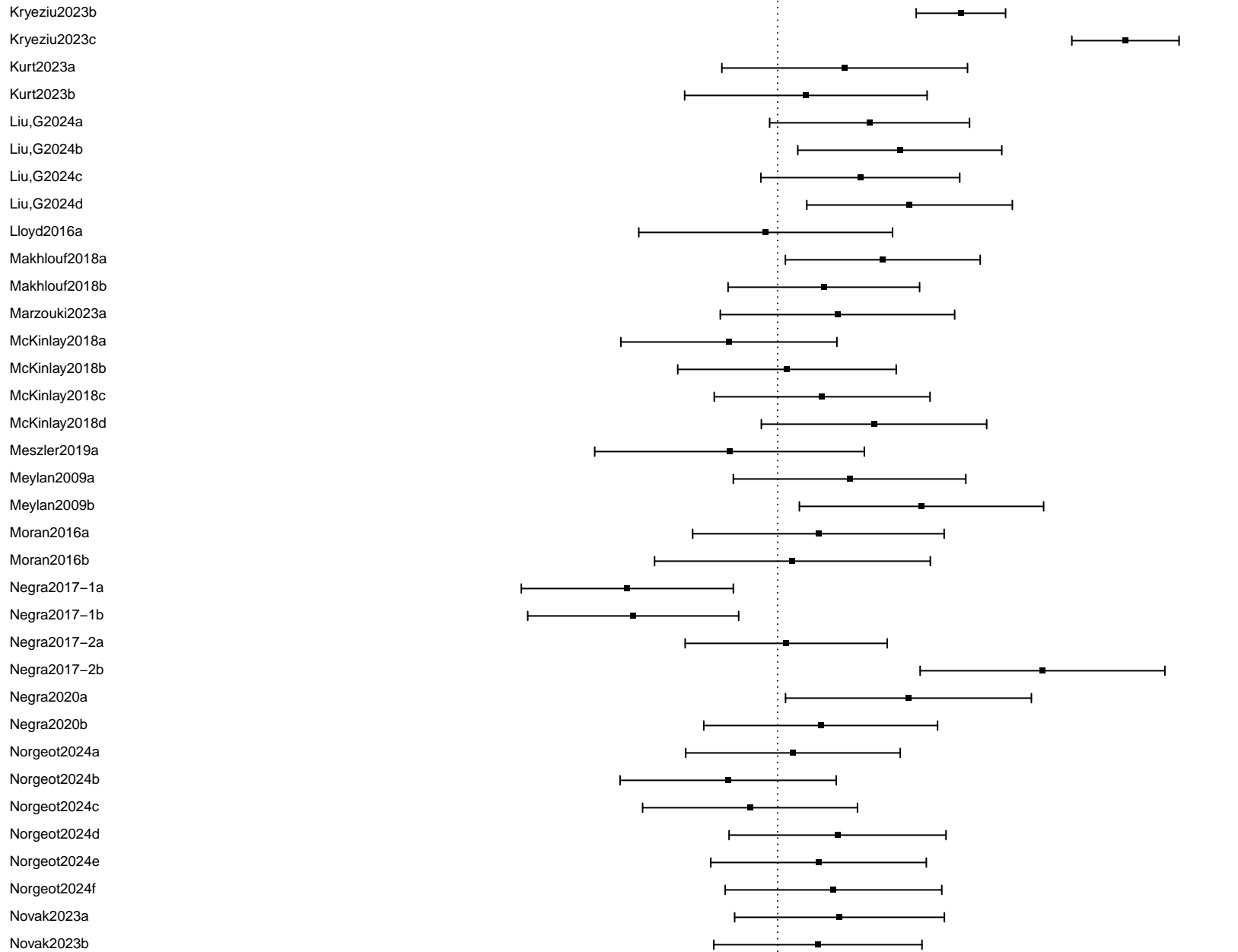

Overall Effects Model

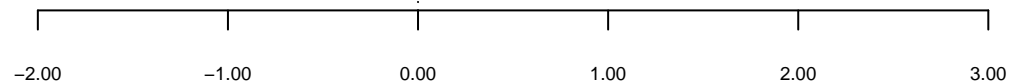

Standardized mean difference (Hedges g)

Supplement: Supplemental Information 20 [file peerj-14-21585-s020.pdf]

Study

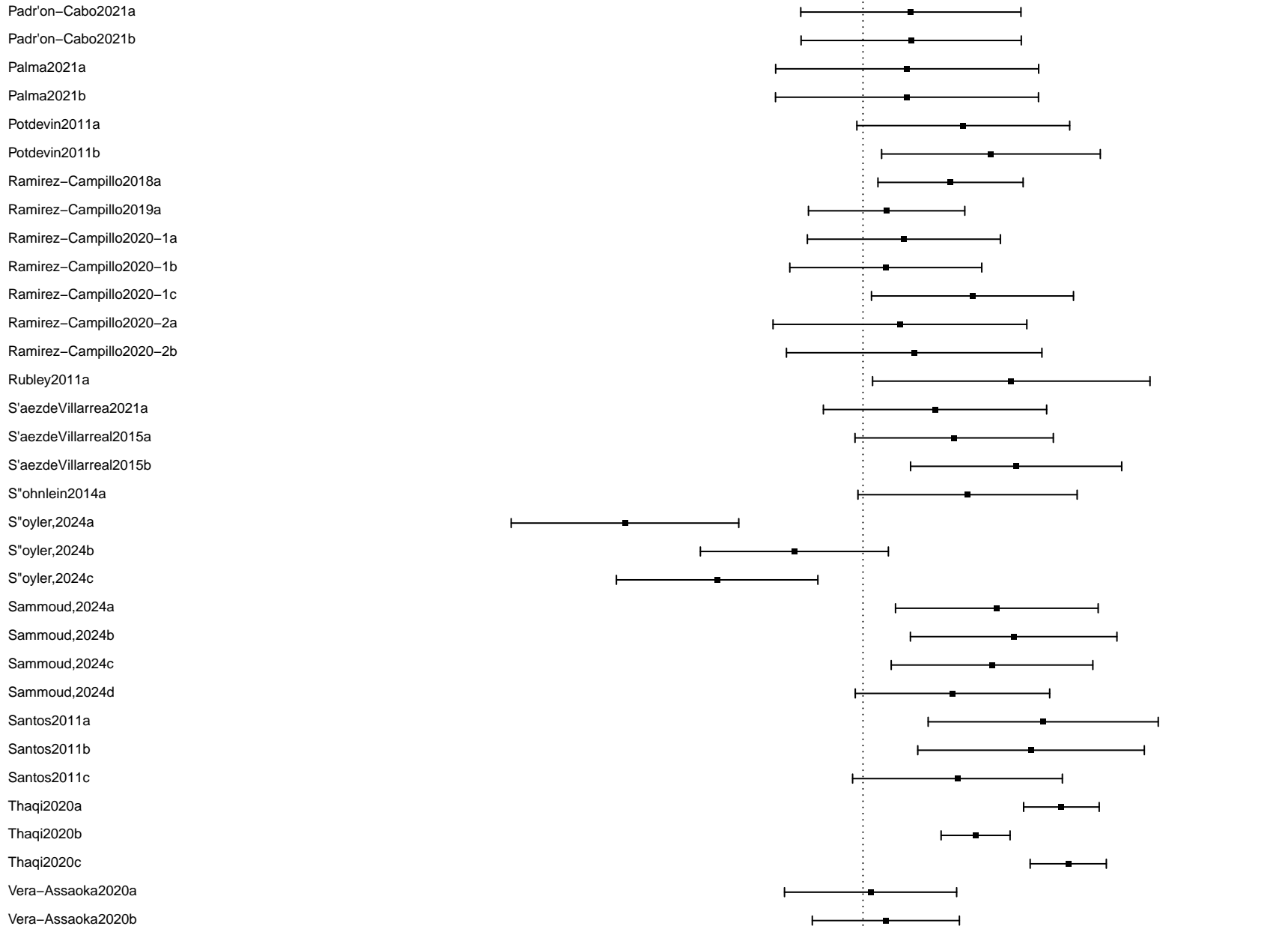

Overall Effects Model

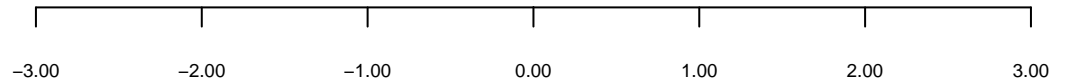

Standardized mean difference (Hedges g)

Supplement: Supplemental Information 21 [file peerj-14-21585-s021.pdf]

## Study

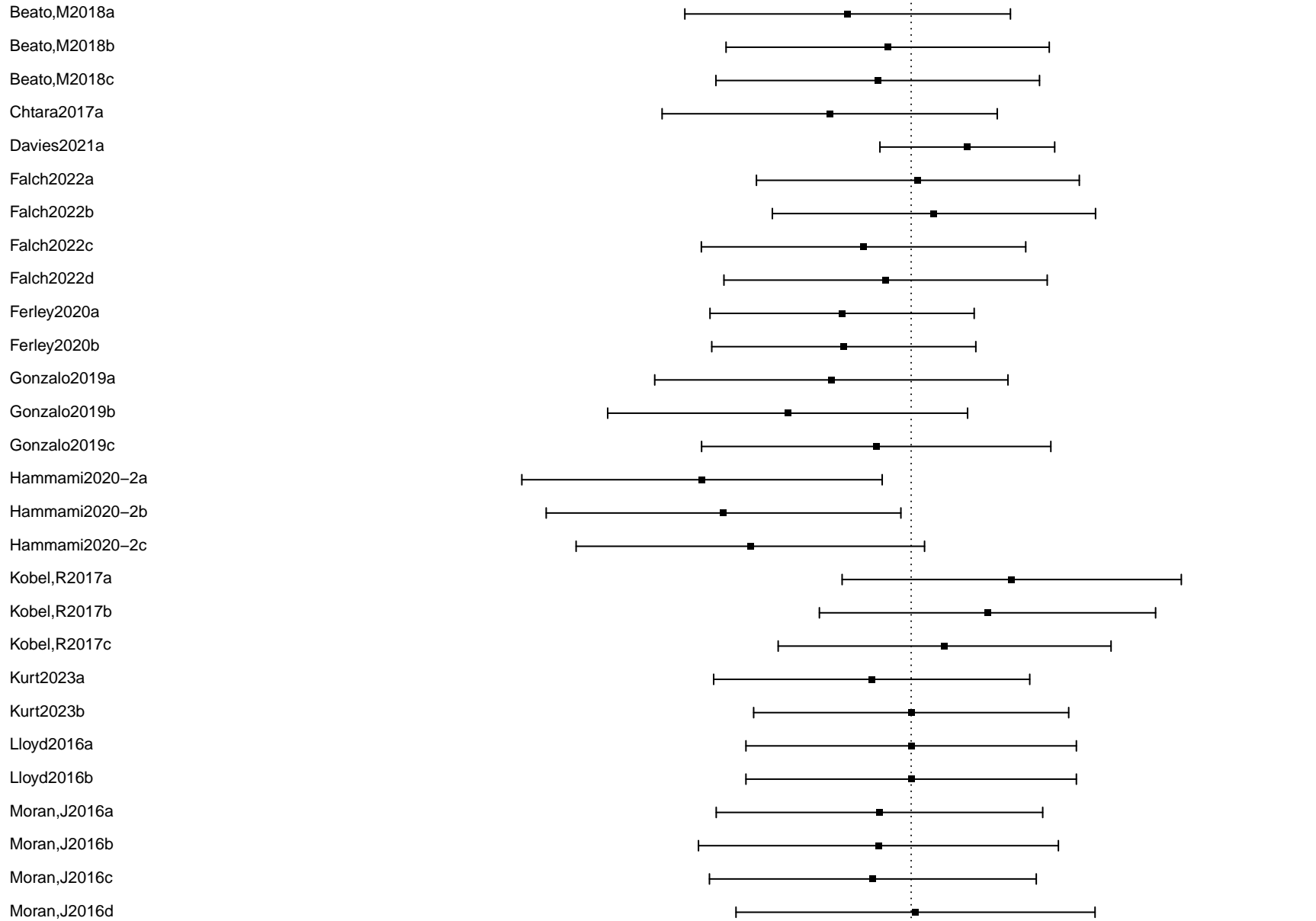

Overall—Effects Model

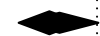

-3.00      -2.00      -1.00      0.00      1.00      2.00

Standardized mean difference (Hedges g)

Supplement: Supplemental Information 22 [file peerj-14-21585-s022.pdf]

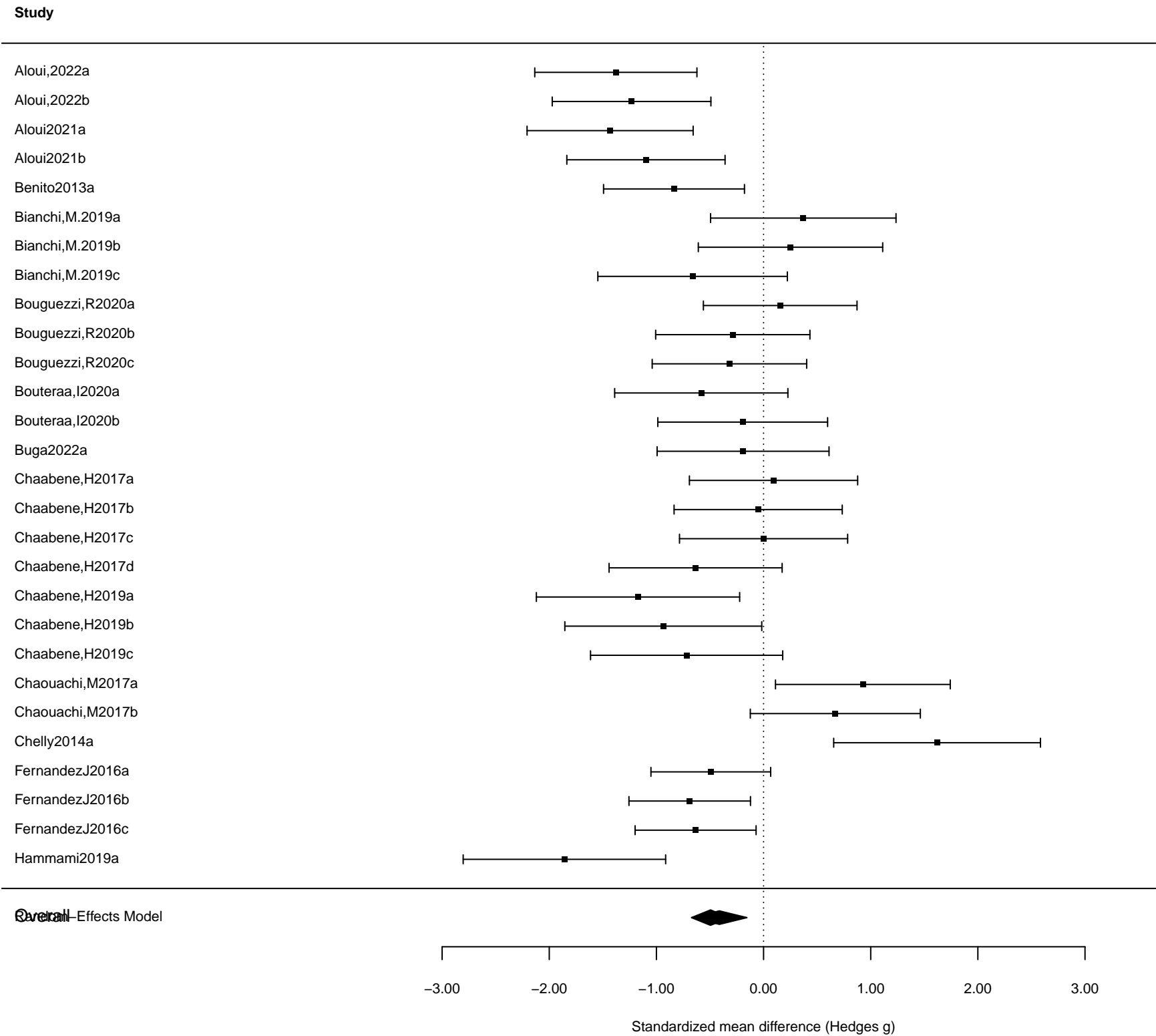

Supplement: Supplemental Information 24 [file peerj-14-21585-s024.pdf]

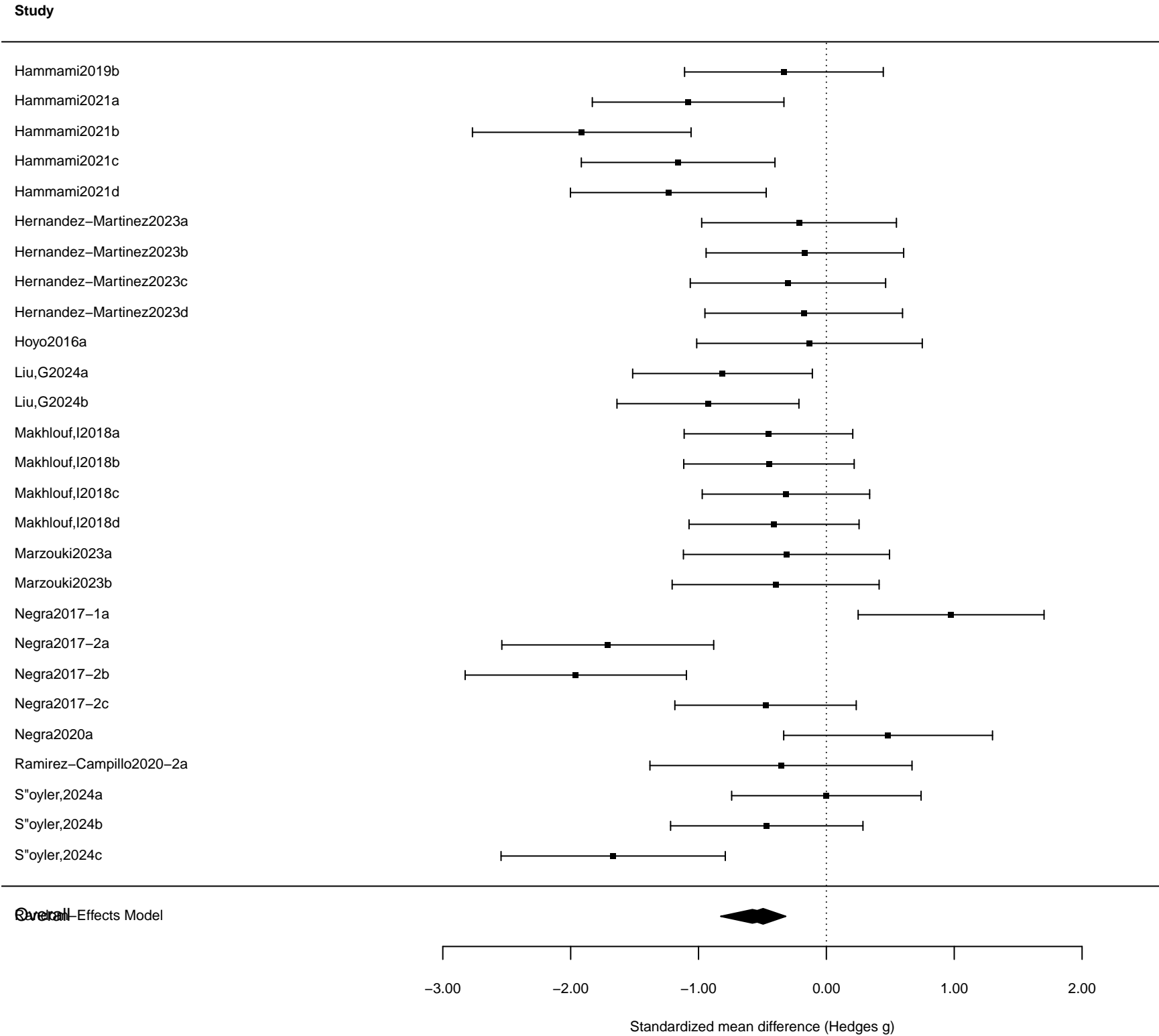

Supplement: Supplemental Information 25 [file peerj-14-21585-s025.pdf]

## Study

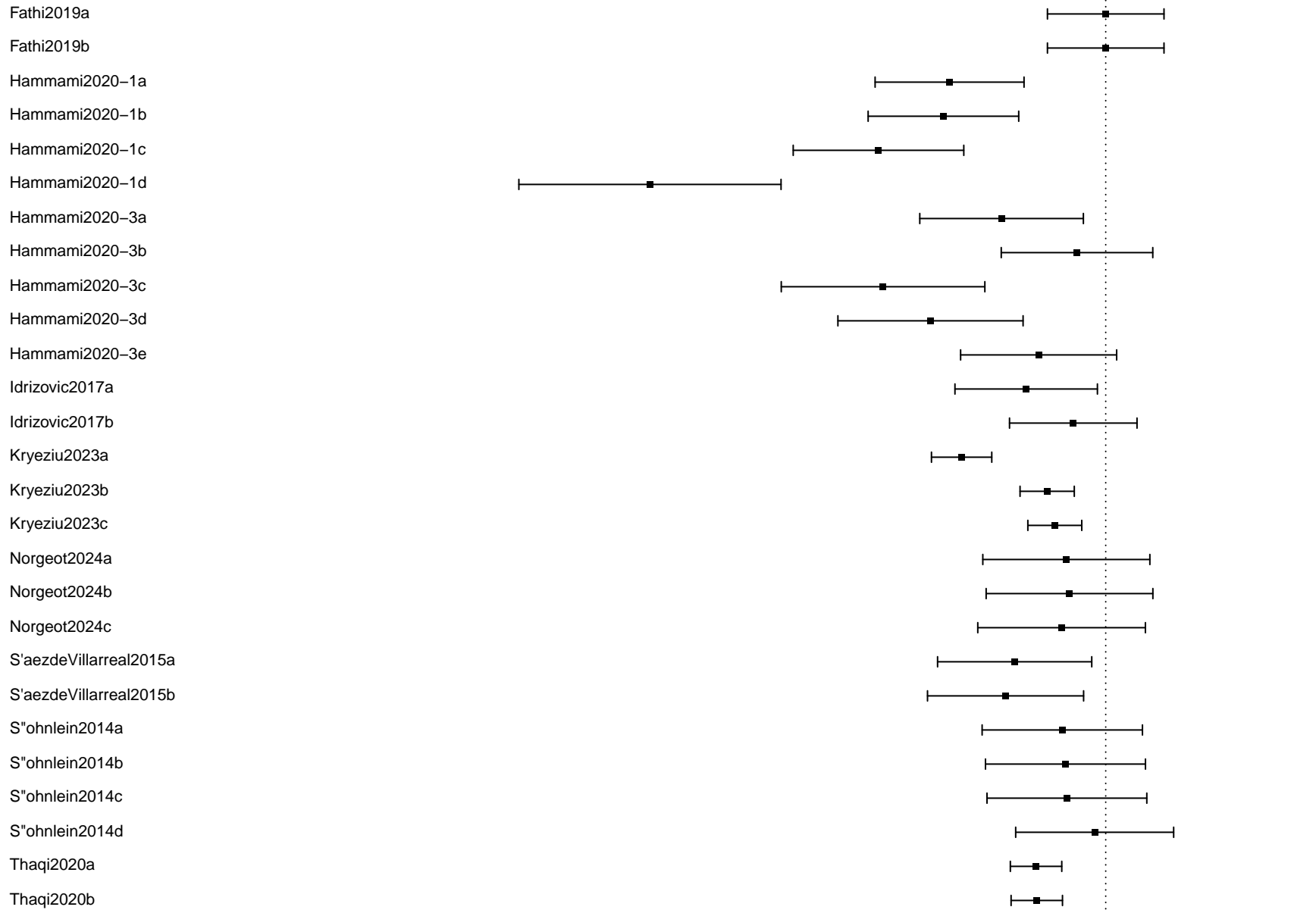

Overall

Effects Model

-8.00 -6.00 -4.00 -2.00 0.00 2.00

Standardized mean difference (Hedges g)

Supplement: Supplemental Information 26 [file peerj-14-21585-s026.pdf]

Study

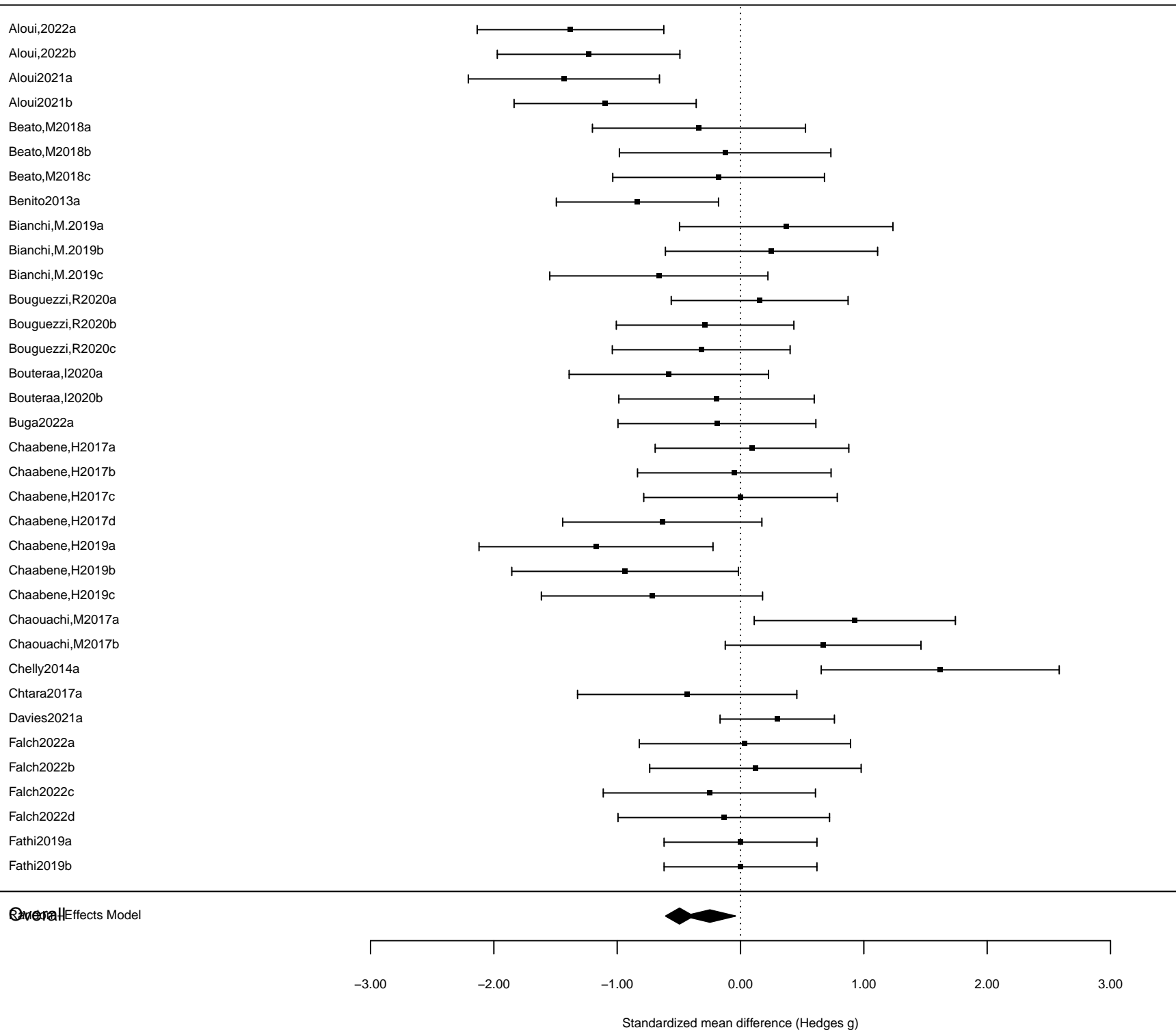

Supplement: Supplemental Information 27 [file peerj-14-21585-s027.pdf]

## Study

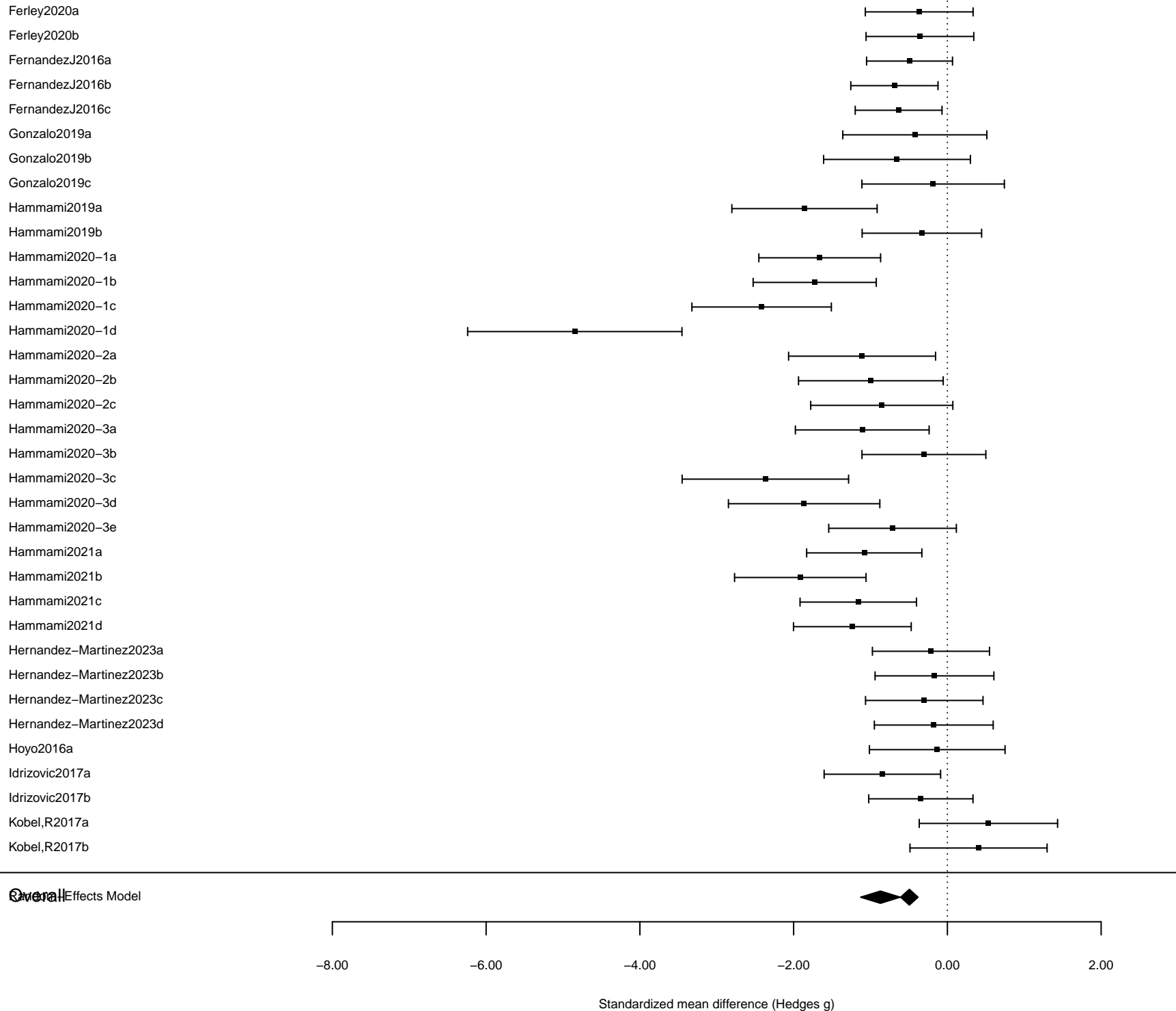

Supplement: Supplemental Information 28 [file peerj-14-21585-s028.pdf]

Study

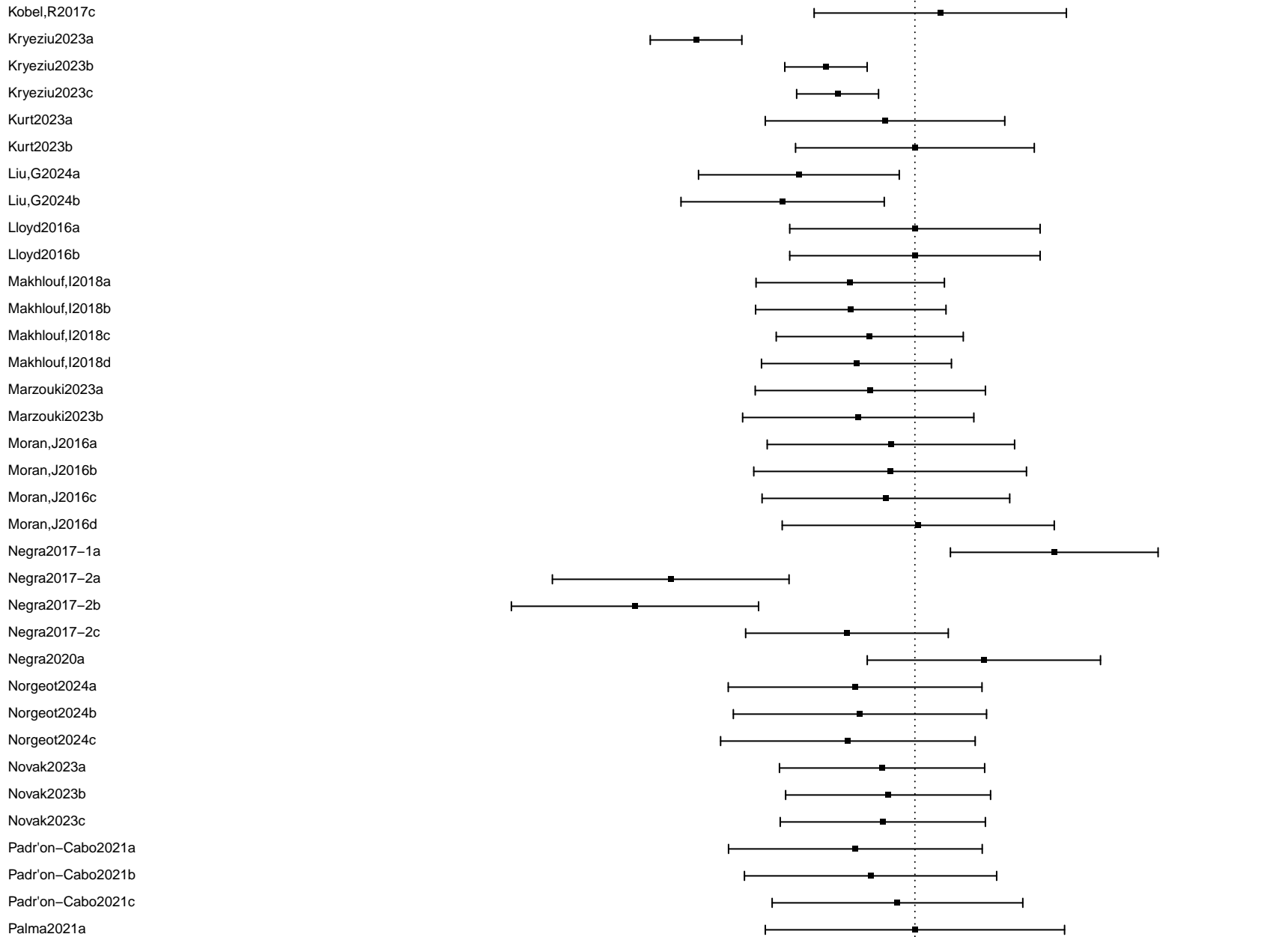

Overall Effects Model

-3.00 -2.00 -1.00 0.00 1.00 2.00

Standardized mean difference (Hedges g)

Supplement: Supplemental Information 29 [file peerj-14-21585-s029.pdf]

Study

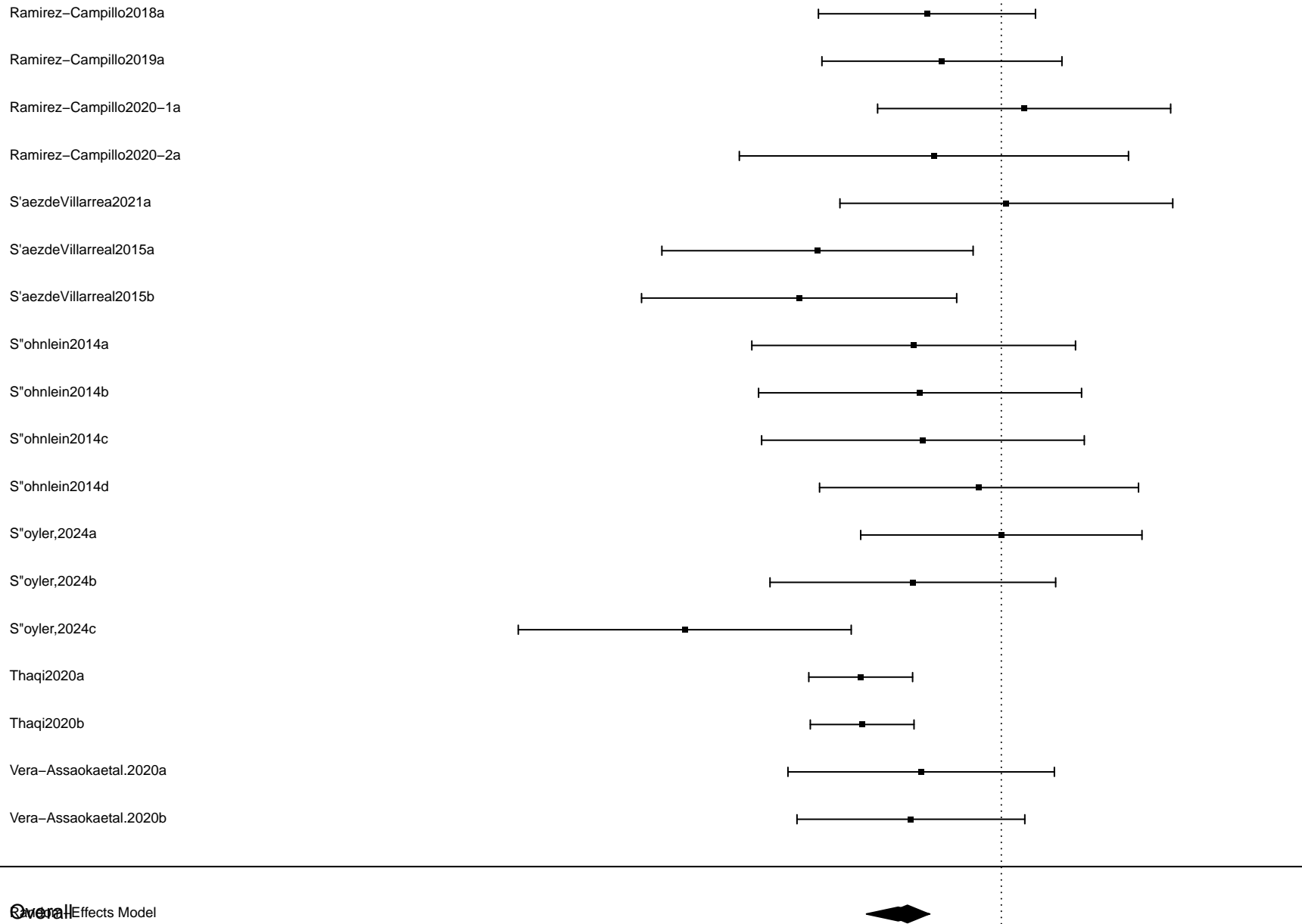

Overall Effects Model

-3.00 -2.00 -1.00 0.00 1.00

Standardized mean difference (Hedges g)

Supplement: Supplemental Information 30 [file peerj-14-21585-s030.pdf]

## Risk of bias domains

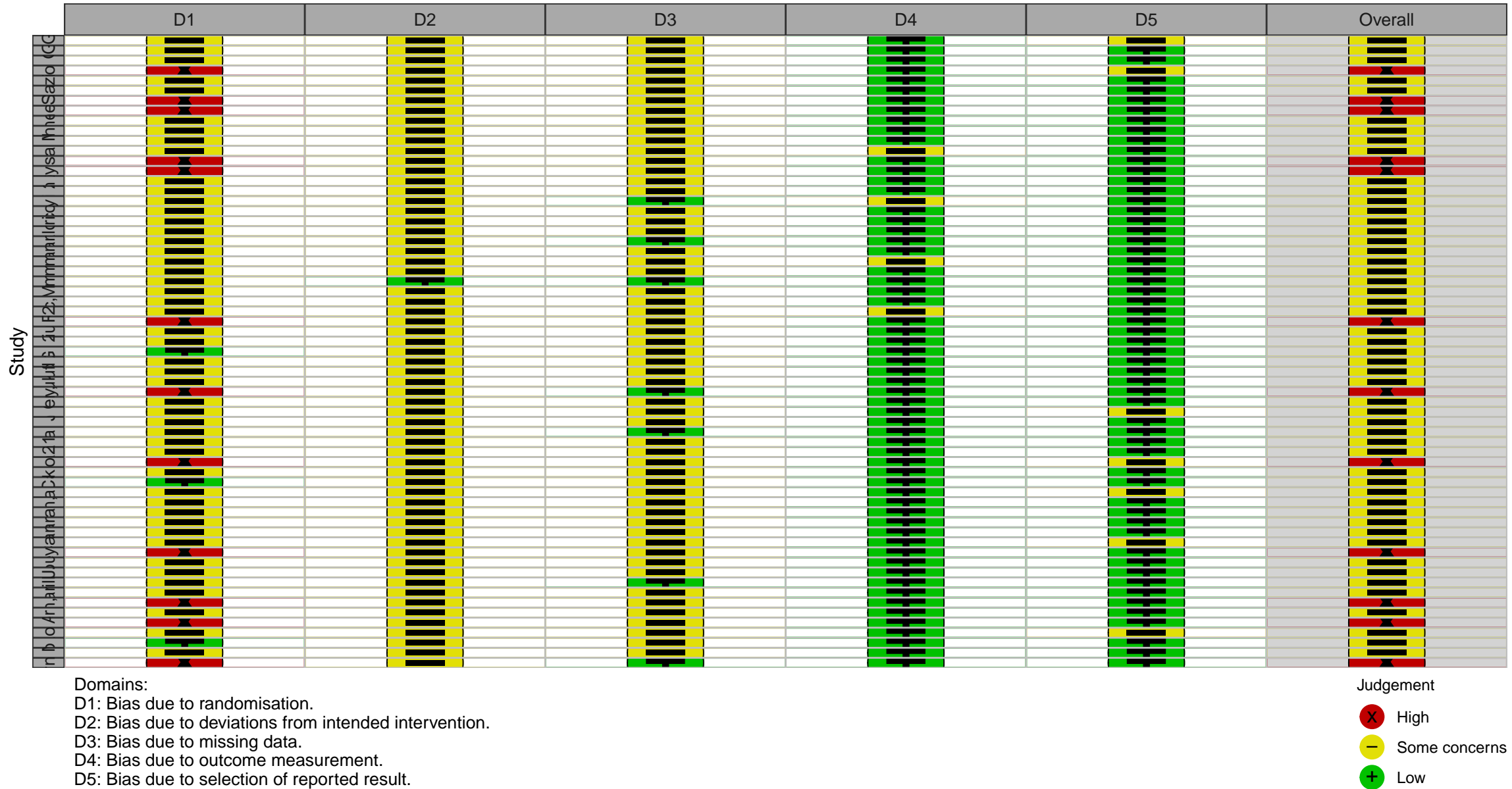

Supplement: Supplemental Information 32 [file peerj-14-21585-s032.pdf]
