# Supplementary material for: The effects of plyometric training on adolescent sports performance: a systematic review and meta-analysis
Source: PeerJ. 2026 Jul 23;14:e21585. doi: 10.7717/peerj.21585 (PMC13401847; doi:10.7717/peerj.21585)
Supplement: Supplemental Information 3 [file peerj-14-21585-s003.docx]

| Outcome | Prespecified hierarchy | SMD | 95% CI | P | I² |
| --- | --- | --- | --- | --- | --- |
| Jump | CMJ > SJ > SLJ | 0.59 | 0.40 to 0.77 | <0.001 | 74% |
| Sprint | 10 m > 20 m > 30 m | -0.40 | -0.59 to -0.20 | <0.001 | 73% |
| Agility | 505 COD test > T-test > COD speed test | -0.54 | -0.71 to -0.36 | <0.001 | 16% |

Supplementary File 3 One-effect-size-per-study sensitivity analyses for jump, sprint, and agility performance

Note: CMJ = countermovement jump; SJ = squat jump; SLJ = standing long jump; COD = change of direction.
