# Supplementary material for: The effects of plyometric training on adolescent sports performance: a systematic review and meta-analysis
Source: PeerJ. 2026 Jul 23;14:e21585. doi: 10.7717/peerj.21585 (PMC13401847; doi:10.7717/peerj.21585)
Supplement: Supplemental Information 4 [file peerj-14-21585-s004.docx]

**Supplementary File 4. Dependency-adjusted three-level and robust variance estimation analyses.**

**Table S4.1. Data structure used in dependency-adjusted analyses**

| **Outcome** | **Effect sizes** | **Study clusters** | **Maximum effects per cluster** | **Missing sport** | **Missing sex** |
| --- | --- | --- | --- | --- | --- |
| Jump | 138 | 63 | 6 | 9 | 2 |
| Sprint | 123 | 52 | 5 | 9 | 2 |
| Agility | 28 | 23 | 4 | 0 | 4 |

Note: Study clusters refer to unique study-level identifiers used in the dependency-adjusted analyses. Multiple effect sizes contributed by the same study cluster were nested within that cluster. Cluster counts may vary across outcomes or moderators because not all studies contributed data to each analysis.

**Table S4.2. Overall dependency-adjusted estimates**

| **Outcome** | **Effect sizes** | **Study clusters** | **SMD** | **95% CI** | **P value** | **Study-level variance** | **Effect-size-level variance** |
| --- | --- | --- | --- | --- | --- | --- | --- |
| Jump | 138 | 63 | 0.613 | 0.447 to 0.779 | <0.001 | 0.324 | 0.037 |
| Sprint | 123 | 52 | -0.414 | -0.583 to -0.245 | <0.001 | 0.271 | 0.034 |
| Agility | 28 | 23 | -0.595 | -0.796 to -0.394 | <0.001 | 0.072 | 0.000 |

**Table S4.3. Duration-based dependency-adjusted subgroup estimates**

| **Outcome** | **Duration subgroup** | **Effect sizes** | **Study clusters** | **SMD** | **95% CI** | **P value** | **df** |
| --- | --- | --- | --- | --- | --- | --- | --- |
| Jump | 6-7 weeks | 37 | 22 | 0.427 | 0.241 to 0.613 | <0.001 | 20.500 |
| Jump | 8 weeks | 75 | 30 | 0.507 | 0.259 to 0.755 | <0.001 | 28.332 |
| Jump | 10-26 weeks | 26 | 11 | 1.243 | 0.818 to 1.668 | <0.001 | 9.535 |
| Sprint | 6-7 weeks | 41 | 19 | -0.194 | -0.347 to -0.040 | 0.016 | 17.506 |
| Sprint | 8 weeks | 55 | 24 | -0.419 | -0.704 to -0.133 | 0.006 | 22.216 |
| Sprint | 10-26 weeks | 27 | 9 | -0.824 | -1.314 to -0.334 | 0.005 | 7.928 |
| Agility | 6-7 weeks | 13 | 12 | -0.487 | -0.751 to -0.222 | 0.002 | 9.503 |
| Agility | 8 weeks | 10 | 9 | -0.612 | -0.952 to -0.273 | 0.004 | 7.389 |
| Agility | 10-26 weeks | 5 | 2 | -1.170 | -4.005 to 1.665 | 0.120 | 1.000 |

**Table S4.4. Main dependency-adjusted moderator analyses**

| **Outcome** | **Moderator** | **Comparison** | **β / difference in SMD** | **95% CI** | **P value** | **df** | **Effect sizes** | **Study clusters** |
| --- | --- | --- | --- | --- | --- | --- | --- | --- |
| Jump | Continuous duration | Per additional intervention week | 0.052 | -0.041 to 0.145 | 0.176 | 3.031 | 138 | 63 |
| Jump | Weekly frequency | Per additional session/week | -0.052 | -0.338 to 0.234 | 0.684 | 7.626 | 138 | 63 |
| Jump | Duration category | 8 weeks vs 6-7 weeks | 0.080 | -0.224 to 0.384 | 0.597 | 42.841 | 138 | 63 |
| Jump | Duration category | 10-26 weeks vs 6-7 weeks | 0.816 | 0.379 to 1.253 | <0.001 | 20.263 | 138 | 63 |
| Jump | Sex | Female vs male | 0.083 | -0.413 to 0.579 | 0.725 | 14.299 | 136 | 62 |
| Sprint | Continuous duration | Per additional intervention week | -0.042 | -0.092 to 0.009 | 0.080 | 2.996 | 123 | 52 |
| Sprint | Weekly frequency | Per additional session/week | -0.066 | -0.305 to 0.172 | 0.526 | 6.482 | 123 | 52 |
| Sprint | Duration category | 8 weeks vs 6-7 weeks | -0.225 | -0.541 to 0.091 | 0.157 | 37.444 | 123 | 52 |
| Sprint | Duration category | 10-26 weeks vs 6-7 weeks | -0.630 | -1.104 to -0.156 | 0.012 | 16.862 | 123 | 52 |
| Sprint | Sex | Female vs male | -0.326 | -1.111 to 0.458 | 0.366 | 8.170 | 121 | 51 |
| Agility | Continuous duration | Per additional intervention week | -0.041 | -0.085 to 0.002 | 0.054 | 1.367 | 28 | 23 |
| Agility | Weekly frequency | Per additional session/week | 0.056 | -0.274 to 0.386 | 0.614 | 2.772 | 28 | 23 |
| Agility | Duration category | 8 weeks vs 6-7 weeks | -0.126 | -0.523 to 0.272 | 0.512 | 15.598 | 28 | 23 |
| Agility | Duration category | 10-26 weeks vs 6-7 weeks | -0.683 | -2.100 to 0.734 | 0.145 | 1.579 | 28 | 23 |

**Table S4.5. Sport-type moderator analyses**

| **Outcome** | **Comparison** | **β / difference in SMD** | **95% CI** | **P value** | **df** | **Effect sizes** | **Study clusters** |
| --- | --- | --- | --- | --- | --- | --- | --- |
| Jump | Basketball vs soccer | 0.001 | -0.408 to 0.409 | 0.997 | 9.621 | 129 | 58 |
| Jump | Volleyball vs soccer | 0.129 | -0.994 to 1.252 | 0.706 | 2.391 | 129 | 58 |
| Jump | Handball vs soccer | 0.864 | 0.202 to 1.526 | 0.017 | 8.145 | 129 | 58 |
| Jump | Tennis vs soccer | 0.042 | -1.917 to 2.001 | 0.865 | 1.125 | 129 | 58 |
| Jump | Hockey vs soccer | -0.283 | -0.598 to 0.031 | 0.066 | 3.589 | 129 | 58 |
| Jump | Swimming vs soccer | 0.433 | 0.219 to 0.647 | <0.001 | 32.193 | 129 | 58 |
| Sprint | Basketball vs soccer | 0.150 | -0.204 to 0.503 | 0.327 | 5.054 | 114 | 47 |
| Sprint | Volleyball vs soccer | 0.115 | -0.579 to 0.809 | 0.599 | 2.457 | 114 | 47 |
| Sprint | Handball vs soccer | -0.411 | -1.518 to 0.697 | 0.410 | 7.038 | 114 | 47 |
| Sprint | Tennis vs soccer | -0.043 | -2.080 to 1.995 | 0.874 | 1.158 | 114 | 47 |
| Sprint | Hockey vs soccer | 0.236 | -0.130 to 0.602 | 0.126 | 2.668 | 114 | 47 |
| Agility | Basketball vs soccer | 0.139 | -1.029 to 1.307 | 0.465 | 1.197 | 28 | 23 |
| Agility | Handball vs soccer | -0.727 | -1.875 to 0.420 | 0.085 | 1.193 | 28 | 23 |
| Agility | Tennis vs soccer | 0.156 | -1.050 to 1.363 | 0.477 | 1.297 | 28 | 23 |
