# Supplementary material for: The effects of plyometric training on adolescent sports performance: a systematic review and meta-analysis
Source: PeerJ. 2026 Jul 23;14:e21585. doi: 10.7717/peerj.21585 (PMC13401847; doi:10.7717/peerj.21585)

Agility (by_dur2_10−26 weeks) page 1/1

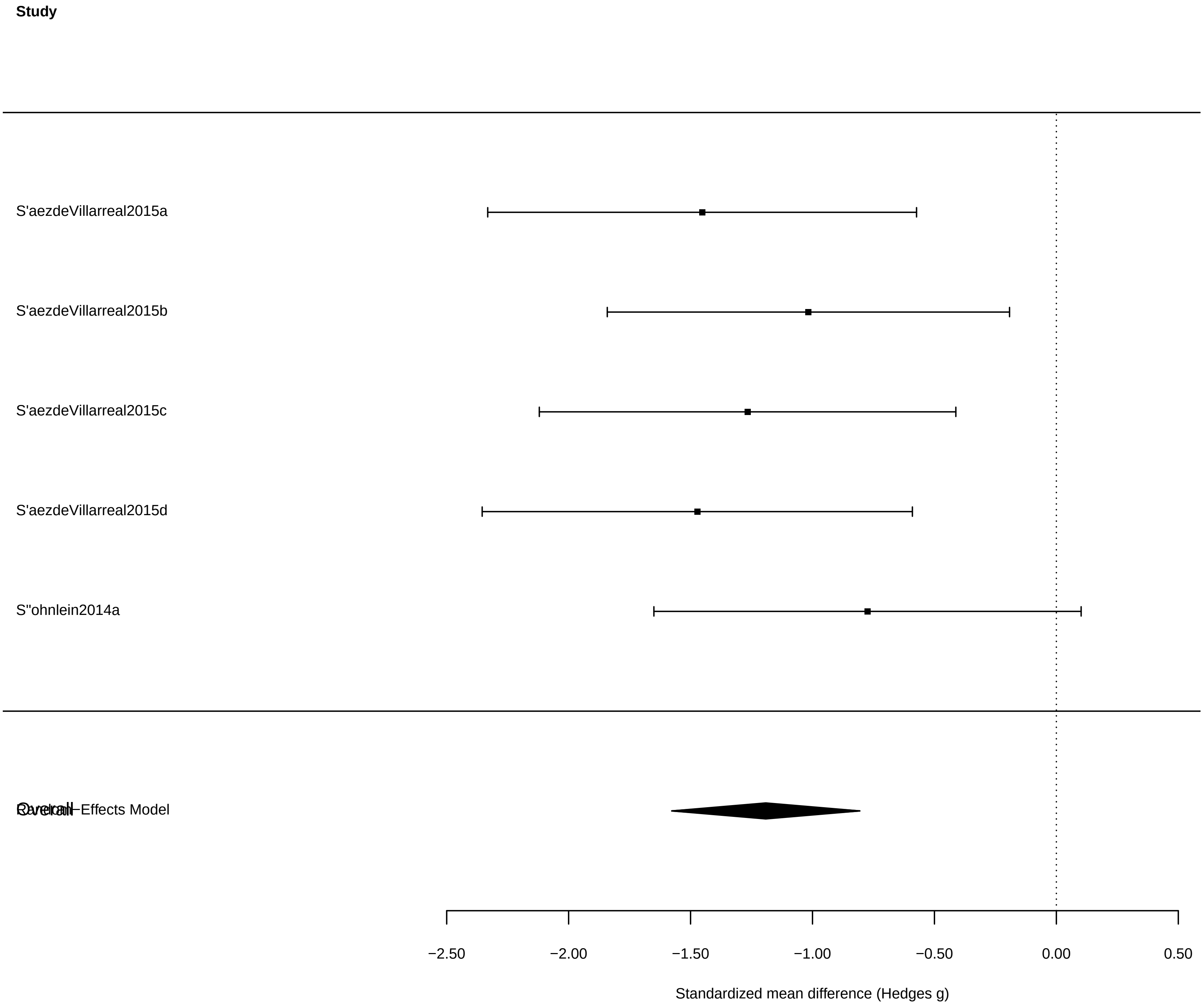

Supplement: Supplemental Information 9 [file peerj-14-21585-s009.pdf]
