# Supplementary material for: The effects of plyometric training on adolescent sports performance: a systematic review and meta-analysis
Source: PeerJ. 2026 Jul 23;14:e21585. doi: 10.7717/peerj.21585 (PMC13401847; doi:10.7717/peerj.21585)
Supplement: Supplemental Information 12 [file peerj-14-21585-s012.pdf]

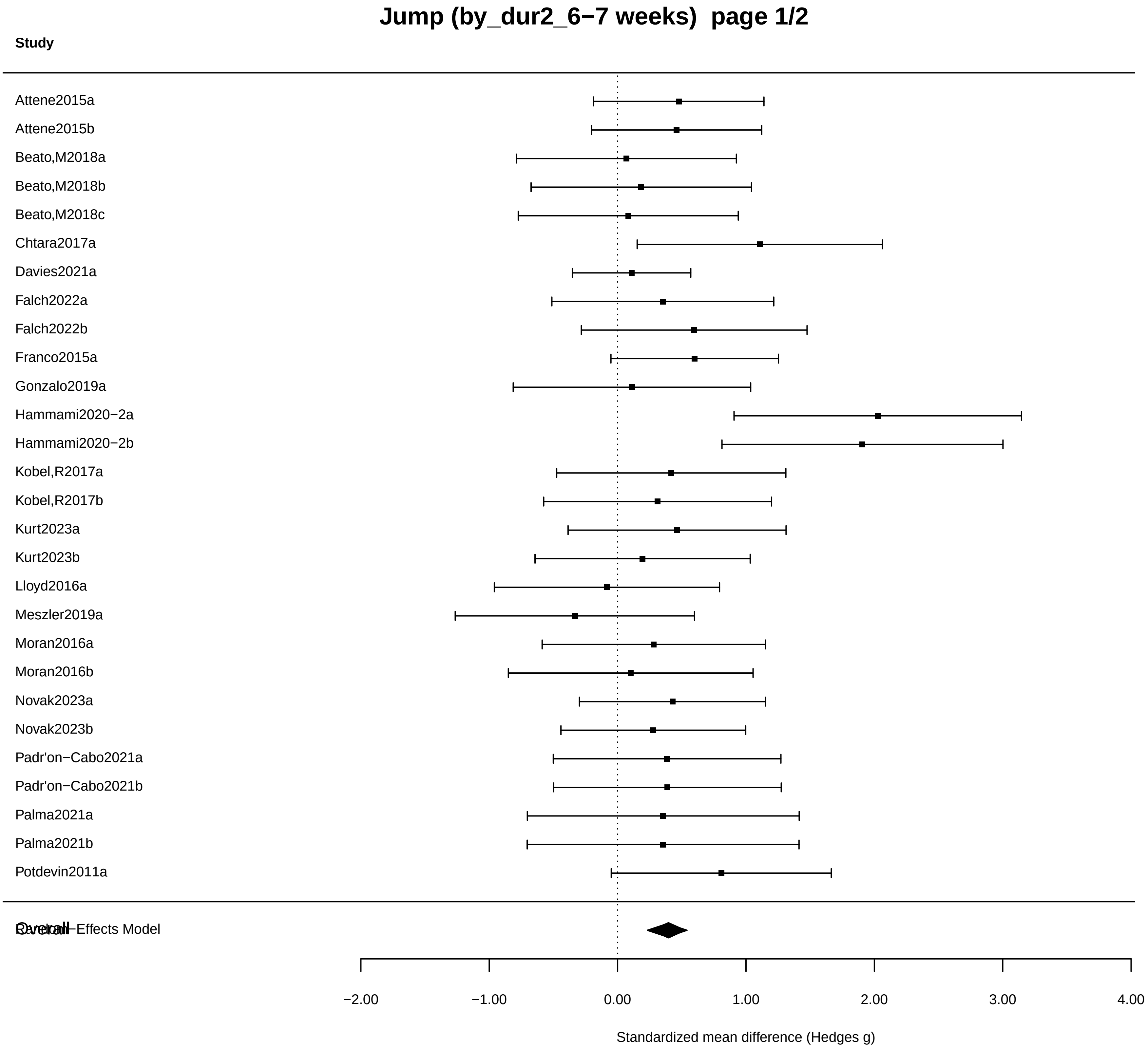

Jump (by_dur2_6−7 weeks) page 1/2
Study
Attene2015a
Attene2015b
Beato,M2018a
Beato,M2018b
Beato,M2018c
Chtara2017a
Davies2021a
Falch2022a
Falch2022b
Franco2015a
Gonzalo2019a
Hammami2020−2a
Hammami2020−2b
Kobel,R2017a
Kobel,R2017b
Kurt2023a
Kurt2023b
Lloyd2016a
Meszler2019a
Moran2016a
Moran2016b
Novak2023a
Novak2023b
Padr'on−Cabo2021a
Padr'on−Cabo2021b
Palma2021a
Palma2021b
Potdevin2011a
Overall
Random−Effects Model
−2.00
−1.00
0.00
1.00
2.00
3.00
4.00
Standardized mean difference (Hedges g)
