# Supplementary material for: The effects of plyometric training on adolescent sports performance: a systematic review and meta-analysis
Source: PeerJ. 2026 Jul 23;14:e21585. doi: 10.7717/peerj.21585 (PMC13401847; doi:10.7717/peerj.21585)

Sprint (by_dur2_6−7 weeks) page 2/2

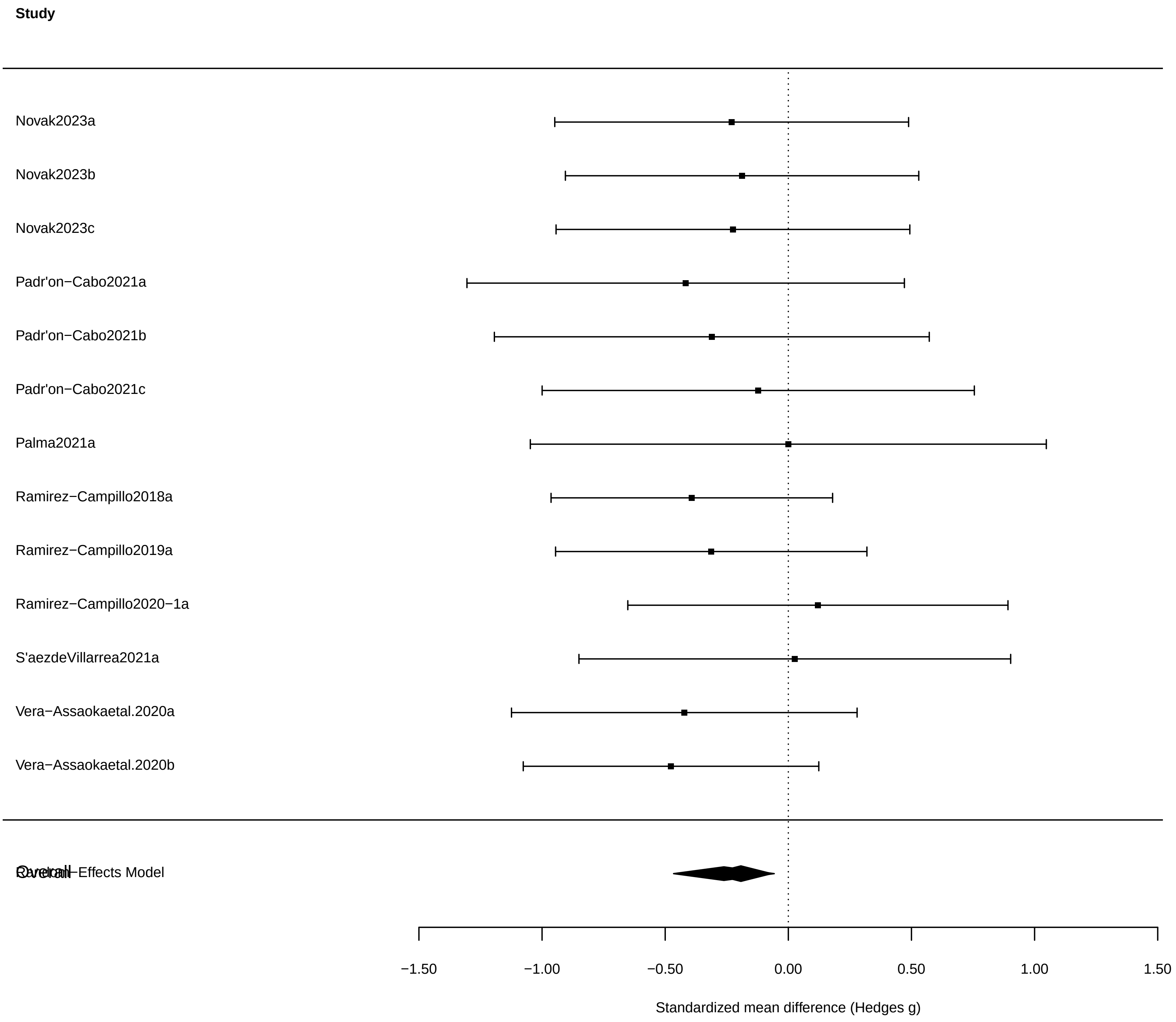

Supplement: Supplemental Information 23 [file peerj-14-21585-s023.pdf]
